# Illuminating Host-Parasite Interaction at the Cellular and Subcellular Levels with Infrared Microspectroscopy

**DOI:** 10.3390/cells11050811

**Published:** 2022-02-25

**Authors:** Hany M. Elsheikha, Alaa T. Al-Sandaqchi, Mohammad S. R. Harun, Francesca Winterton, Ali Altharawi, Nashwa A. Elsaied, Carl W. Stevenson, William MacNaughtan, John G. M. Mina, Paul W. Denny, Gianfelice Cinque, Ka Lung Andrew Chan

**Affiliations:** 1 School of Veterinary Medicine and Science, University of Nottingham, Loughborough LE12 5RD, UK; alaa_tariq2001@yahoo.com (A.T.A.-S.); fran88@protonmail.com (F.W.); nashw95@gmail.com (N.A.E.); 2 Faculty of Veterinary Medicine, University of Liège, Sart Tilman B43, B-4000 Liège, Belgium; 3 School of Biosciences, University of Nottingham, Loughborough LE12 5RD, UK; carl.stevenson@nottingham.ac.uk (C.W.S.); billmacnaughtan@gmail.com (W.M.); 4 Department of Biomedical Science, Advanced Medical & Dental Institute, Universiti Sains Malaysia, Bertam, Kepala Batas, Pulau Pinang 13200, Malaysia; mosyamsulre@usm.my; 5 Department of Pharmaceutical Chemistry, College of Pharmacy, Prince Sattam Bin Abdulaziz University, Al-Kharj 11942, Saudi Arabia; a.altharawi@psau.edu.sa; 6 Institute of Pharmaceutical Science, King’s College London, London SE1 9NH, UK; 7 Department of Biosciences, University of Durham, Durham DH1 3LE, UK; j.g.m.mina@durham.ac.uk (J.G.M.M.); p.w.denny@durham.ac.uk (P.W.D.); 8 Diamond Light Source, Harwell Science and Innovation Campus, Didcot OX11 0DE, UK; gianfelice.cinque@diamond.ac.uk

**Keywords:** *Toxoplasma gondii*, host-pathogen interaction, blood-brain barrier, vibrational spectroscopy, synchrotron infrared microspectroscopy

## Abstract

*Toxoplasma gondii* (*T. gondii*) is an opportunistic protozoan that can cause brain infection and other serious health consequences in immuno-compromised individuals. This parasite has a remarkable ability to cross biological barriers and exploit the host cell microenvironment to support its own survival and growth. Recent advances in label-free spectroscopic imaging techniques have made it possible to study biological systems at a high spatial resolution. In this study, we used conventional Fourier-transform infrared (FTIR) microspectroscopy and synchrotron-based FTIR microspectroscopy to analyze the chemical changes that are associated with infection of human brain microvascular endothelial cells (hBMECs) by *T. gondii* (RH) tachyzoites. Both FTIR microspectroscopic methods showed utility in revealing the chemical alterations in the infected hBMECs. Using a ZnS hemisphere device, to increase the numerical aperture, and the synchrotron source to increase the brightness, we obtained spatially resolved spectra from within a single cell. The spectra extracted from the nucleus and cytosol containing the tachyzoites were clearly distinguished. RNA sequencing analysis of *T. gondii*-infected and uninfected hBMECs revealed significant changes in the expression of host cell genes and pathways in response to *T. gondii* infection. These FTIR spectroscopic and transcriptomic findings provide significant insight into the molecular changes that occur in hBMECs during *T. gondii* infection.

## 1. Introduction

Nearly one-third of the world’s human population are chronically infected by the intracellular protozoan parasite *Toxoplasma gondii* [1]. This protozoan can cause serious illness, especially in immunocompromised patients and in pregnant women [2,3]. *T. gondii* can also result in significant economic losses in production animals, particularly in sheep and goats. While some treatments are available, they are limited in scope [3]. Therefore, more understanding of parasite pathogenesis and host response mechanisms will lead to the development of novel therapeutics capable of protecting against disease caused by *T. gondii* infection of humans as well as many economically vital farm animal species.

How a cell responds to *T. gondii* infection and, in turn, how *T. gondii* attempts to moderate that response have been a topic of increasing interest. One such response of the parasite is to alter the host cell metabolism [4]. *T. gondii* is an obligate intracellular organism and auxotrophic for several key metabolites. For these reasons, *T. gondii* depends on the host’s metabolism to leverage carbon and nutrient sources in order to sustain its growth [4]. Various analytical approaches have been used to characterize the metabolic changes that occur in host cells and tissues in response to *T. gondii* infection. For example, mass-spectrometry-coupled with liquid chromatography has been used to characterize the impact of *T. gondii* infection on the metabolism of cerebral cortex [5], cerebellum [5] and the whole brain in mice [6]. Nuclear magnetic resonance (NMR) was used to study the metabolic changes in human cerebrovascular endothelial cells during *T. gondii* infection [7]. Raman microspectroscopy has been used to characterize the spatio-temporal molecular changes in human retinal cells infected by *T. gondii* tachyzoites [4].

Vibrational infrared (IR) spectroscopy is a powerful label-free optical method that has been widely used to interrogate the chemical composition of biological materials [8]. This spectroscopic approach detects the absorbance of IR light due to molecular vibrations, thereby generating a chemical fingerprint that contains (semi)quantitative information on the constituent molecules, such as nucleic acids, proteins, lipids, and carbohydrates. Therefore, it provides an excellent opportunity to investigate biochemical changes in biological samples (tissues, cells, cell organelles) under external conditions, such as infection and drug treatment [9]. Recent advances in IR spectroscopy, including the development of cutting edge instrumentation and chemometric analytical approaches, provide more efficient tools for faster and more precise characterization of biological materials [10]. The IR spectroscopic method is often combined with optical microscopes for scanning of large areas with high spatial resolution at the cellular and sub-cellular level [11].

Synchrotron based Fourier-transform IR (FTIR) microspectroscopy has been used to characterize chemical changes in host cells under attack by *T. gondii* [9]. Another study has reported the use of two ZnS hemispheres that significantly enhance the spatial resolution and resolve subcellular features such as the nucleolus of single cells [12]. Compared to CaF_2_ hemispheres [13,14], the lower end of the spectral range using ZnS hemispheres is extended, from 1200 cm^−1^ to below 1000 cm^−1^, a significant improvement allowing the carbohydrate and DNA bands in that region to be studied [12]. This ZnS hemisphere device offers scope to characterize *T. gondii* interaction with host cells by FTIR at a spatial resolution that has never been observed before. To achieve subcellular spatial resolution, a recent study has shown that synchrotron IR source with a small aperture (~2.7 μm × 2.7 μm) and smaller step size (~0.9 μm) can produce the best imaging result in terms of image sharpness and spectral quality [15].

The aim of this study was to identify the biochemical signatures that underpin the cellular response to *T. gondii* infection. The combined synchrotron source with the ZnS hemispheres can provide the sub-cellular resolution needed for the extraction of FTIR spectra from specific regions in infected host cells. Results obtained by the ZnS hemisphere device and the synchrotron source were compared with data obtained using conventional FTIR microspectroscopy. In addition, high-throughput RNA sequencing (RNA-seq) technology was used to determine the transcriptome profiles and differentially expressed genes in *T. gondii*-infected and uninfected cells.

## 2. Materials and Methods

### 2.1. Cell Lines and Culture Conditions

Human brain microvascular endothelial cells (hBMECs) were grown in Gibco^®^ Roswell Park Memorial Institute (RPMI) 1640 media (Thermo Fisher Scientific, Waltham, MA, USA) as described previously [9,16,17,18,19]. African Green Monkey (*Cercopithecus aethiops*) kidney epithelial (Vero) cells (ECACC, HPACC, Salisbury, UK) grown in Dulbecco’s Modified Eagle Medium (DMEM) were used as surrogate host cells to sustain the growth of *T. gondii* tachyzoites. Both cell lines were maintained in monolayers in T75 (75 cm^2^) NUNC™ tissue culture flasks (Fisher Scientific, Leicestershire, UK) in humidified 5% CO_2_-95% air at 37 °C. All cells used in subsequent experiments had >99% viability as determined by the trypan blue exclusion assay.

### 2.2. Parasite Culture and Purification

Tachyzoites of *T. gondii* RH strain were maintained in Vero cell monolayers grown in NUNC™ T75 tissue culture flasks, as described previously [20]. The parasites were collected when lysis of at least 70% of Vero cell monolayers, due to infection, was detected. The parasite culture was maintained by inoculating the content of the infected culture flasks into new Vero cell monolayers as described previously [9,19]. To separate the tachyzoites from the host cell debris, we used PD-10 desalting columns filled with Sephadex-25 gel filtration material (GE Healthcare, Amersham, UK), as previously described [21]. Freshly purified tachyzoites were used to infect hBMECs at a multiplicity of infection (MOI) of 2 (i.e., 2 tachyzoites: 1 host cell).

### 2.3. FTIR Micro Spectroscopy Analysis

The hBMECs were seeded on 13 mm Ø x 1 mm sterilized CaF_2_ windows (Crystran Ltd., Dorset, UK) positioned in the bottom of 24-well tissue culture plastic plates and incubated at 37 °C with 5% CO_2_. After 24 h, *T. gondii* tachyzoites were added to the cell monolayers. The infected cells were incubated for 24 and 48 h post infection (hpi). At each time point the medium was removed and cells (infected and control) were gently washed twice with chilled 1× Phosphate Buffered Saline (PBS; pH 7.2) to remove traces of culture medium. Cells were fixed in 4% paraformaldehyde (PFA) for 10 min followed by gentle rinsing in ultrapure water 18.2 MΩ cm (Direct-Q 3 UV, Millipore), and air-dried for ~30 min. At least 10 independent spectra from 10 cells were recorded in each sample. FTIR measurements were performed in the transmission mode using a MCT detector fitted to an FTIR microscope (Bruker Hyperion 2000 microscope [Bruker Optics, Coventry, UK]) coupled to an FTIR spectrometer (Bruker Tensor 27 FTIR spectrometer) as described previously [19].

### 2.4. Synchrotron-Based FTIR Micro Spectroscopy

In this experiment, hBMECs (1 × 10^4^) were seeded in 24-well tissue culture plates and incubated at 37 °C with 5% CO_2_ overnight to allow the adherence and growth of the cells. Then, freshly egressed tachyzoites were used to infect hBMEC monolayers. Control hBMECs were mock-infected by medium only, without *T. gondii* infection. At 24 and 48 hpi, cells were harvested by trypsinization, and the trypsinized cell suspension was fixed using 4% PFA in PBS for 10 min. Briefly, the flat surface of the hemisphere was gently rinsed using 1× PBS and fixed cell preparations were then drop-cast on three ZnS hemispheres per time point. The cells were allowed to dry for a few minutes followed by washing using PBS, PBS/ultrapure water and ultrapure water. Finally, two ZnS hemispheres were assembled with a thin (6 μm) film of ultrapure water (~50 µL) sandwiched between the flat surfaces of the two hemispheres. Four cell samples were examined at each time point after infection.

The measurements were carried out at the Diamond Light Source synchrotron facility, (B22 beamline, MIRIAM). The imaging system (Hyperion 3000, Bruker Optics) includes 36× reverse Cassegrain reflective objective and condenser (Numerical aperture = 0.5 in air, increased to 1.12 through the ZnS with a refractive index 2.25), a mercury cadmium telluride (MCT) liquid nitrogen cooled single element detector 50 µm in size coupled with a Vertex 80 v spectrometer. An aperture size of ~2.7 μm × 2.7 μm and a step size of ~0.9 μm was used at 4 cm^−1^ spectral resolution and 128 scans (~20 s) per spectrum.

### 2.5. Spectral Data Processing

FTIR images and the extracted spectra were preprocessed using OPUS software (OPUS 7.8, Bruker Optics). FTIR images were produced using the peak integration method whereby a straight baseline between a defined range was used to calculate the area under the absorbance curve. 7–10 extracted spectra from different regions of the cells (4 cells from each condition) were first cut to a 3000–1000 cm^−1^ spectral region followed by concave rubber band baseline correction with 3 iterations and 64 baseline points. Spectra were then vector normalized. Pair-wised principal component analysis (PCA) was performed on the extracted spectra using PyChem software (http://pychem.sourceforge.net/; accessed on 6 December 2021) [22]. The analysis was focused on two spectral regions of interest: 3000–2800 cm^−1^ and 1800–1000 cm^−1^. Significance differences between the averaged extracted spectra from each region within the cell (control against infected) was determined using a *T*-test *p* value < 0.05 calculated using Microsoft^®^ Excel 2010.

### 2.6. Transcriptomic Analysis

RNA-Seq was used to comprehensively study the changes in gene expression in host cells in response to *T. gondii* infection. Two biological replicates of control and *T. gondii*-infected hBMECs at 6, 24 and 48 hpi were prepared for mRNA sequencing analysis. Briefly, the total RNA was isolated from the cell lysate of control and infected hBMECs by using a Qiagen RNeasy Plus Mini kit (Qiagen, Hilden, Germany). The quality and quantity of the extracted RNA was determined using the Agilent^®^ RNA 6000 Nano kit and 2100 Bioanalyser (Agilent Technologies LDA UK Limited, Cheshire, UK), respectively. All samples with RQN (RNA quality number) values of 9.0 or above were processed for sequencing. Messenger RNA (mRNA) libraries were prepared using Illumina’s TruSeq Stranded mRNA HT Kit [RS-122-2103] (Illumina EMEA, Cambridge, UK). Sequencing was performed as 150 bp paired end reads on Illumina’s HiSeq4000 platform (Illumina EMEA, Cambridge, UK) according to Illumina specifications. mRNA sequencing was performed by the Oxford Genomics Centre, The Wellcome Trust Centre for Human Genetics, University of Oxford, United Kingdom.

RNA-seq data analysis was carried out using VEuPathDB Galaxy Site (https://veupathdb.globusgenomics.org/; accessed on 6 December 2021); an interactive web-based platform for large-scale data analysis. The quality of RNA sequences was checked by FastQC Galaxy version 0.11.3. Trimmomatic Galaxy Version 0.36.5) and low-quality reads (default settings) and adapters (ILLUMINACLIP—trim standard adapter sequence for paired ended for MiSeq and HiSeq sequencers) were removed. The cleaned reads were aligned to the HostDB-52_HsapiensREF_Genome using HISAT2 (Galaxy Version 2.0.5). The read counting was performed using HTSeq-count (Galaxy Version HTSeq: default; SAMTOOLS: 1.2; PICARD: 1.134). We analyzed the differential gene expression level between infected and control samples by using EdgeR (Galaxy Version 1.0.0), with filtered counts used as input [23]. The individual gene expression was calculated as the mean expression of each gene averaged over all samples of each group and presented as the logarithm of counts per million reads. The false-discovery rate (FDR) and adjusted *p* value were used for multiple test comparisons according to the Benjamini-Hochberg procedure. We used a threshold (adjusted *p*-value/FDR value < 0.05 and absolute log_2_FC ≥ 1 or ≤−1) to identify the differentially expressed genes (DEGs). PCA, hierarchical clustering heatmaps and volcano blots of the DEGs at all time points and at each time point post-infection were produced by using PCA plot w ggplot2 (Galaxy Version 2.2.1), heatmap2 (Galaxy Version 3.0.1), and R Studio (version 4.1.1) with ggplot2 package for visualization. To identify the biological relevance of the DEGs, pathway analysis was performed using the REACTOME pathway browser (https://reactome.org/PathwayBrowser/; accessed on 13 December 2021) and single-sample gene set enrichment analysis (ssGSEA) to obtain pathway expression values [24].

## 3. Results

### 3.1. Single Cell Analysis Using Conventional FTIR Microspectroscopy

**Figure 1:**
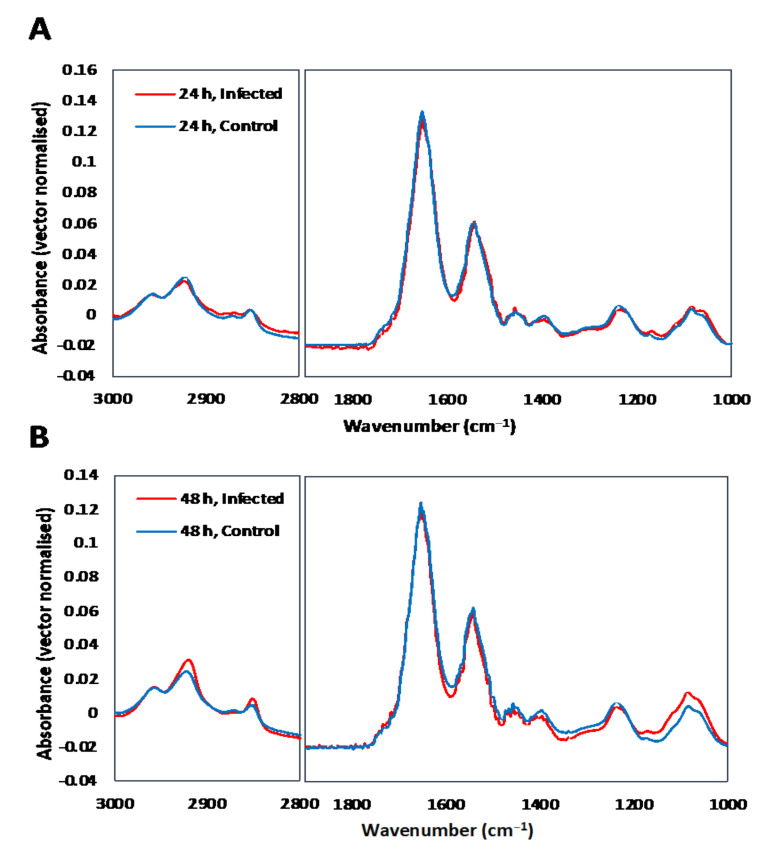

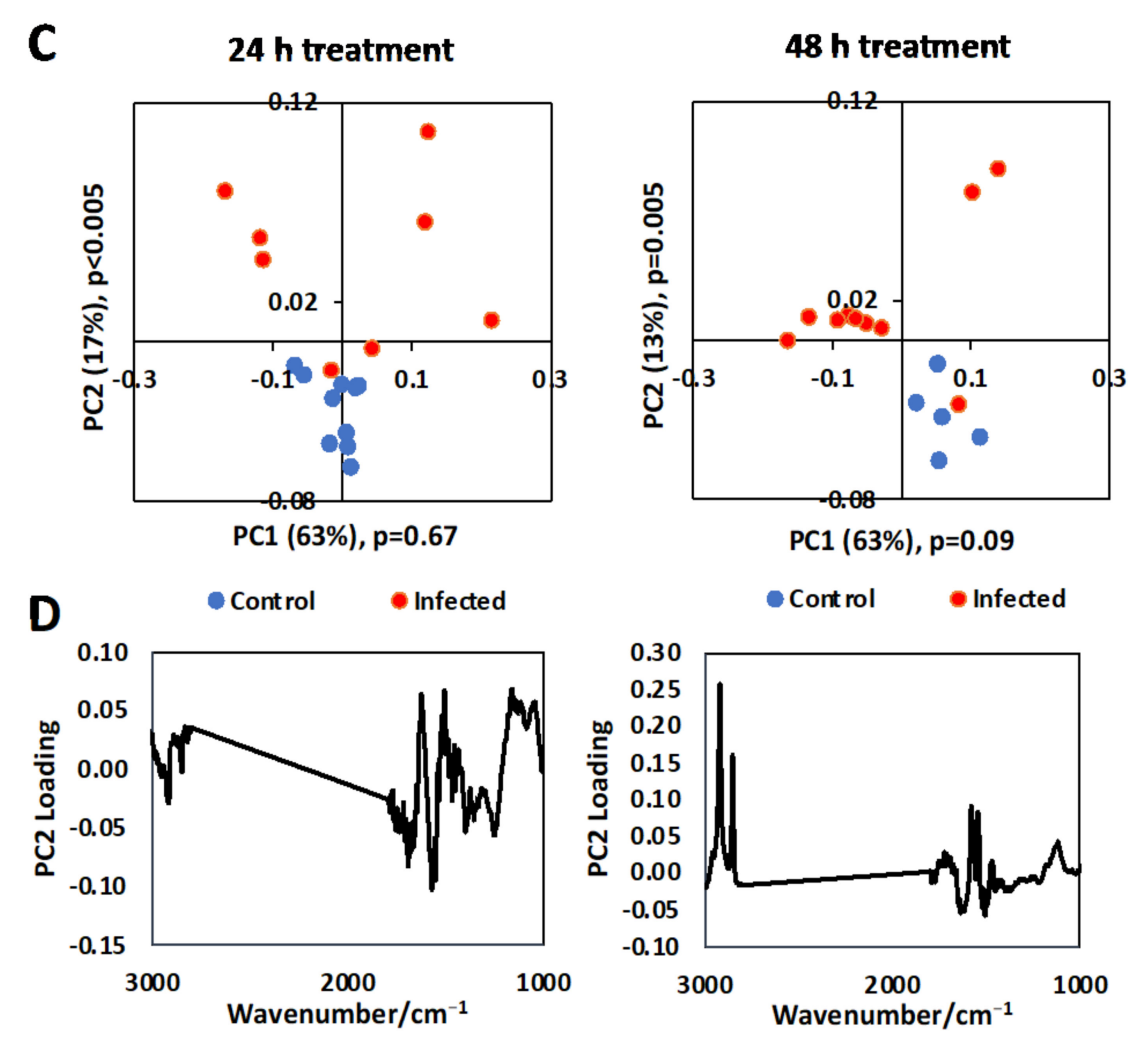
The averaged spectra of cells at 24 h (**A**) and 48 h (**B**) after treatment. Pair-wised (control versus infected) PCA of spectral data obtained from traditional FTIR microscopy at 24 h and 48 h after infection with (**C**) showing the score scatter plots and (**D**) showing the PC2 loading vectors.

The spectra and PCA results obtained from the single cell analysis using a conventional FTIR microscopy without synchrotron or the hemispherical ZnS lenses approach are shown in Figure 1. These results are based on the averaged FTIR absorbance from the individual hBMECs and therefore it was not possible to reveal subcellular differences between the infected and the control cells. Figure 1A,B show that the lipid content is slightly lowered 24 hpi but was increased at 48 hpi. The results of PCA at 24 and 48 hpi (Figure 1C,D) revealed insignificant differences between the control and infected cells from the first principal component (PC1). However, significant differences were observed in PC2 with changes mainly associated with the lipid CH stretching bands and protein amide I and II. Cells infected for 24 h had an overall lower lipid (negative ν(CH) bands) in the loading vector, however at 48 hpi, the lipid content was higher in the infected cells, in agreement with the results shown in Figure 1A,B.

### 3.2. Subcellular Mapping Using Synchrotron MicroFTIR and ZnS Hemispheres

**Figure 2:**
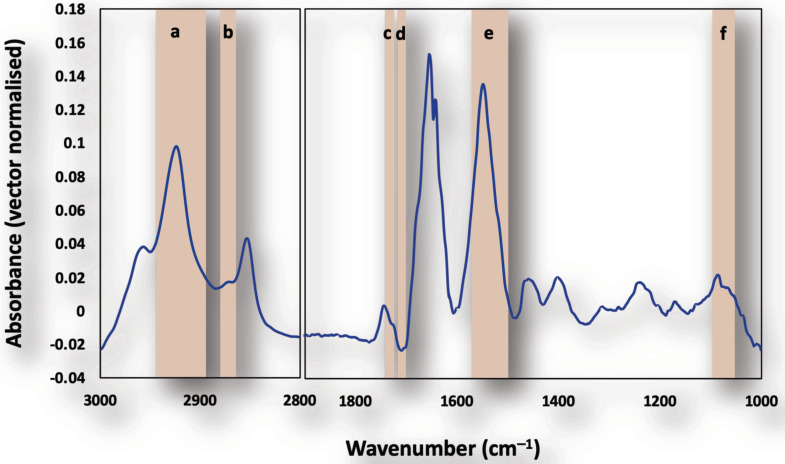
Typical SR-FTIR spectrum (3000–2800 cm^−1^ and 1800–1000 cm^−1^) of the cell measured through the ZnS lenses with peaks a-f used to generate FTIR images of (a) lipid ν_asym_(CH_2_) (2946–2908 cm^−1^), (b) DNA ν_sym_(CH_3_) (2885–2865 cm^−1^), (c) lipid ν(C=O) (1763–1718 cm^−1^), (d) DNA ν(C=O) (1730–1707 cm^−1^), (e) protein amide II (1593–1496 cm^−1^) and (f) phosphate ν_sym_(PO_2_)^-^ (1102–1065 cm^−1^), respectively.

We applied the newly developed hemispherical ZnS lenses to increase the spatial resolution of FTIR imaging so that spectra from nucleus and parasite-containing cytosol regions in single infected cells could be discerned. We also compared the spectral difference between the cytosol of uninfected cells in control culture, uninfected cells in infected culture, and infected cells as a function of infection time. A typical spectrum extracted from the imaging measurements is shown in Figure 2, highlighting the high quality of spectrum obtained despite the small aperture used.

Different bands were used to produce FTIR images that represent the different subcellular areas within a single cell. The overall outline of the cell was marked by the protein amide II (1593–1496 cm^−1^) and phosphate ν_sym_(PO_2_)^−^ (1102–1065 cm^−1^) bands; lipid ν_asym_(CH_2_) (2946–2908 cm^−1^) and ν(C=O) (1763–1718 cm^−1^) bands were used to highlight the lipid-rich cytosol region, ν_sym_(CH_3_) (2885–2865 cm^−1^); while the ν(C=O) (1730–1707) band was used to reveal the area of the nucleus [25]. Cells were measured in their hydrated state (in aqueous environment) to avoid large differences in the microenvironment of analyzed cells. For example, dried nucleic acid can lead to significantly different spectral bands compared to their hydrated counterpart [26]. As a result, amide I at ~1656 cm^−1^ was not used in the image generation because higher noise/saturation was observed in spectral regions where water has strong absorbances (>3000 cm^−1^ and 1680–1620 cm^−1^).

**Figure 3:**
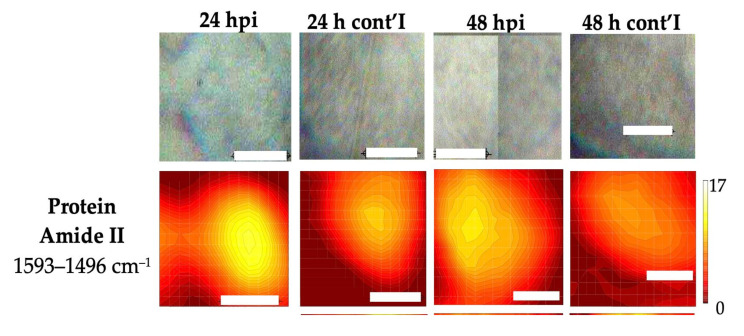

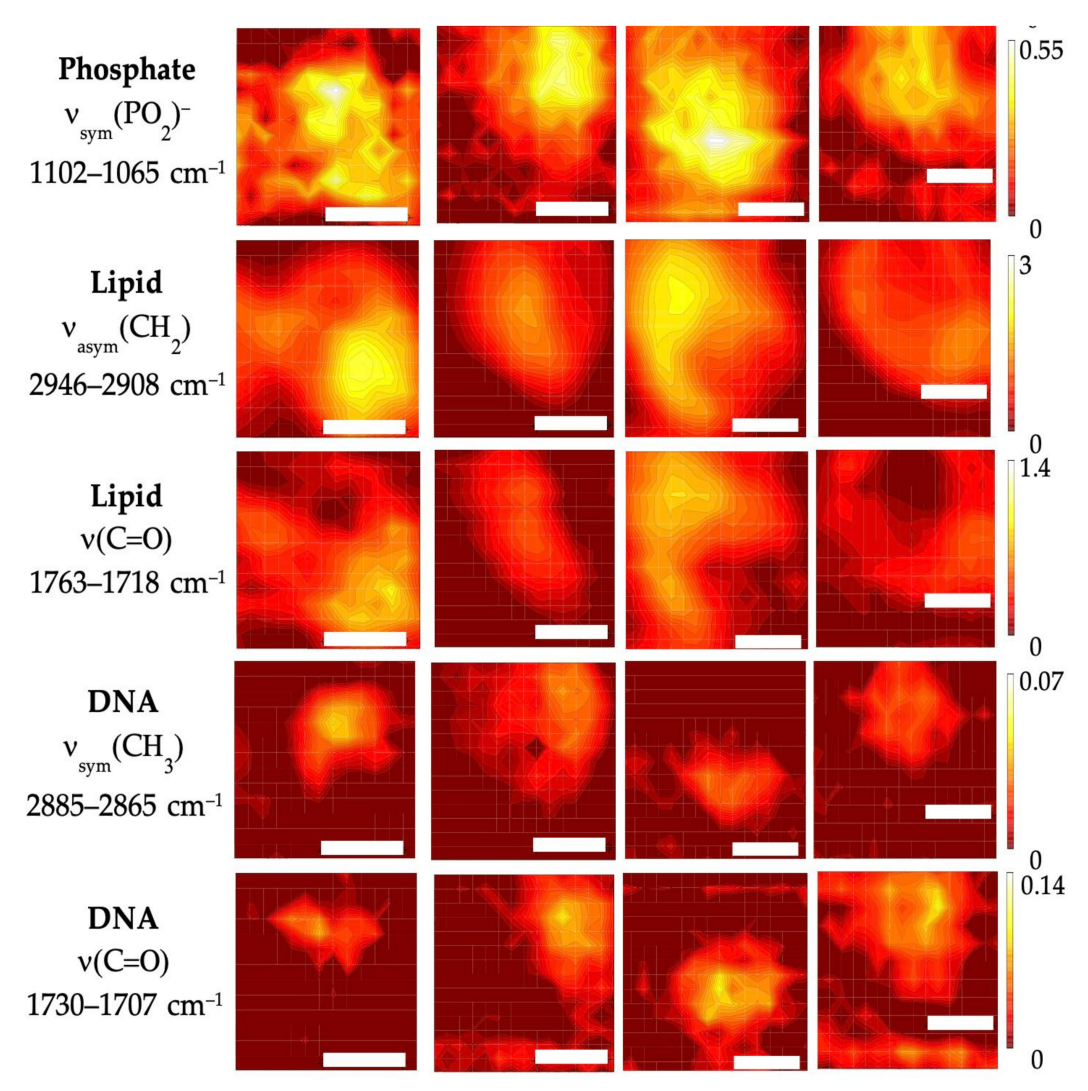
A representative set of visible images (top row) and FTIR images (integration range shown on the left column) of infected and control cells at 24 and 48 hpi. Scale bars = 10 μm.

**Figure 4:**
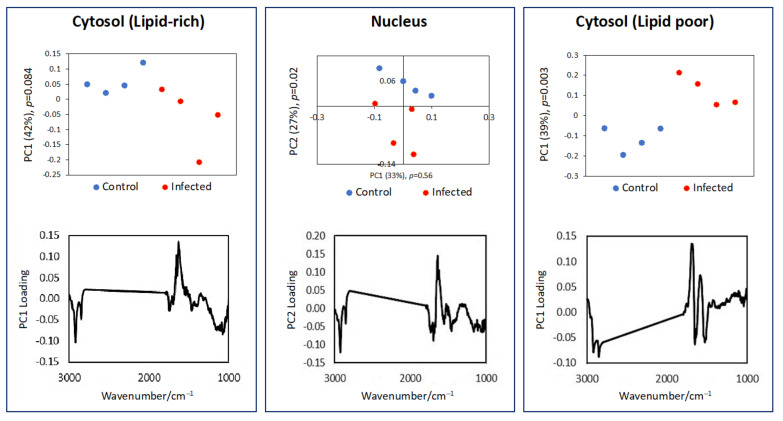
Pairwised (control versus infected) PCA of the extracted spectra (shown in Figure S5) from the three subcellular regions (cytosol (lipid-rich), nucleus, and cytosol (lipid poor)) with the most distinct differences between infected and uninfected cells at 24 hpi.

**Figure 5:**
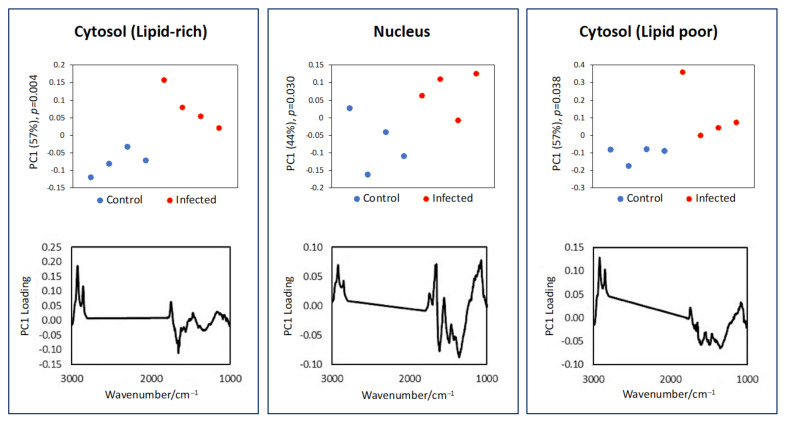
Pairwised (control versus infected) PCA1 of the extracted spectra (shown in Figure S6) from the three subcellular regions (cytosol (lipid-rich), nucleus, and cytosol (lipid poor)) with the most distinct differences between infected and uninfected cells at 48 hpi.

A representative set of FTIR maps of infected and control cells at 24 and 48 h after infection are shown in Figure 3. The images of all analyzed cells are shown in Figures S1–S4. The amide II and ν_sym_(PO_2_)^−^ peak integration produced FTIR maps showing the overall shape of the cell, which correlated well with the visible images. The FTIR maps from the two lipid bands ν_asym_(CH_2_) and ν(C=O) showed uneven lipid distribution within the single cell and appeared to be broadly complementary to the FTIR maps of the DNA ν_sym_(CH_3_) and ν(C=O) bands, which indicated the nuclear region of the cell. Whilst the SR-FTIR approach revealed the distribution of various subcellular components inside a single cell, no obvious differences between the infected and control cells were observed. However, the high spatial resolution approach allowed spectra from different regions of a single cell to be dissociated. The spectra extracted from lipid-rich and lipid-poor cytosol, and nucleus regions were compared using pair-wised PCA to reveal the spectroscopic difference between the control and infected cells. The results of the PCA are shown in Figure 4 and Figure 5.

Figure 4 shows the pair-wised comparison between the control and infected cells at 24 hpi. There is a separation in PC1 (the most important PC) from the lipid-rich cytosol region, but the separation was not significant (*p* > 0.05) and not in any of the other pairwised (control versus infected) comparison PCs. The large range of PC1 score in the infected cell has shown that the infected cells were highly variable in the lipid-rich domain, suggesting a heterogeneous response among the infected cells at the early stage of infection. In the lipid-poor region of the cytosol, PC1 has shown a significant difference between control and infected cells with changes mainly found in the protein amide I and II peaks. In the nucleus region, PC1 did not show significant difference (*p* > 0.05) between the control and infected cells. However, there was a significant difference from PC2 (the second most important PC) with the loading vector highlighting positive bands for the protein amide I, II and III and negative bands for the lipid ν(CH_2_) and ν(C=O) bands, suggesting a higher protein and lower lipid content in the nucleus of the control when compared to the infected cell at 24 hpi. This result is consistent with the result obtained by conventional FTIR microspectroscopy.

Figure 5 shows the results at 48 hpi. In contrast to 24 h, all pair-wised comparisons between control and infected cells showed significant differences (*p* < 0.05) in PC1, suggesting that longer infection time produced more marked effect on the infected cells. Changes in the cytosol, whether lipid-rich or lipid-poor, produced a similar difference as shown by the similarity in their PCA loading plots. The infected cells showed an increase in lipid ν(CH_2_) and ν(C=O) bands. The nucleus region also showed an increase in lipid in the infected cell with rather complex changes in the protein, phosphate, and nucleic acid regions, suggesting several biochemical changes have occurred.

### 3.3. Global Transcriptomic Changes

**Figure 6:**
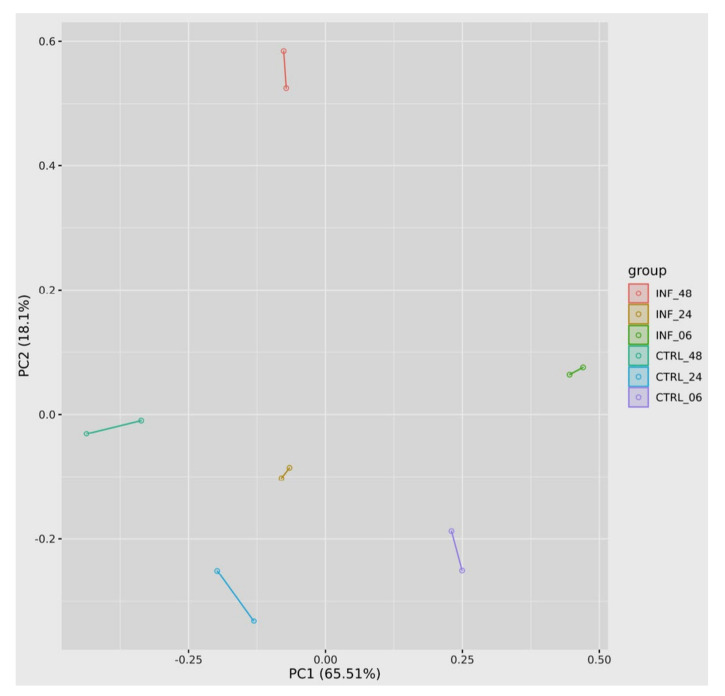
Principal Component Analysis of RNA-seq-based differential gene expression between infected and control hBMECs at 6-, 24- and 48-hpi. Two biological replicates are shown for each time point.

**Figure 7:**
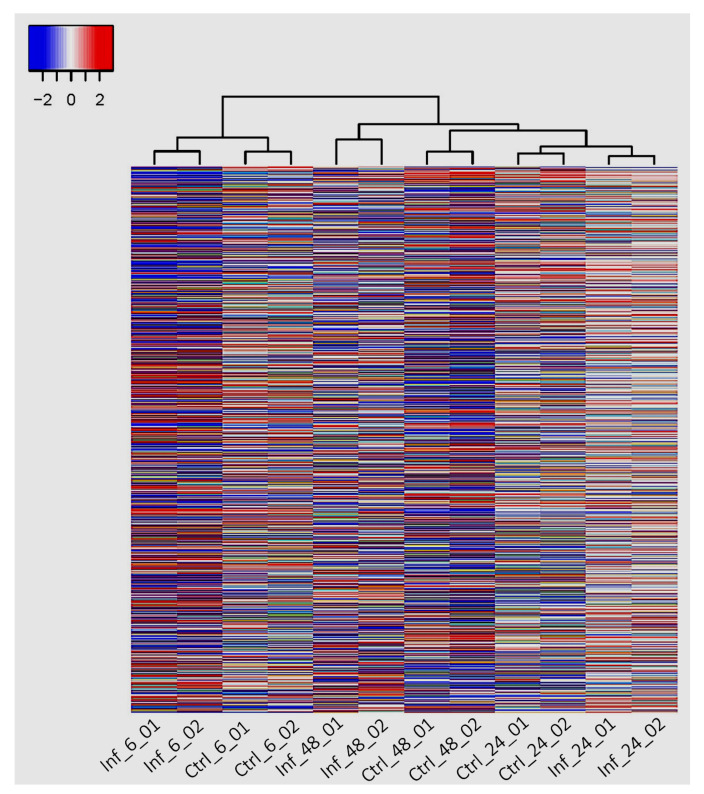
Heatmap showing hierarchical clustering of 533 DEGs at 6-, 24-, and 48-hpi. Only genes with log_2_FC of < 1 or > 1 and FDR < 0.05 were included in the heatmap. The color scale from blue (low) to red (high) corresponds to the gene expression values. Columns represent the samples (infected and control) and time points, while rows represent individual genes. The phylogenetic tree above the heatmap shows the hierarchical clustering of the samples.

RNA sequencing analysis of *T. gondii*-infected and uninfected hBMECs was performed to obtain more insight into the host-parasite relationship with a focus on genes that contribute to the mechanisms of metabolic adaptation and immune response of the host cell to *T. gondii* infection. The overall expression pattern of the DEGs at 6-, 24-, and 48-hpi was visualized using a PCA plot, which shows the variation caused by infection. Comparing control and infected cells at the same time point showed that the largest distance was detected between control and infected samples at 48 hpi, while the shortest distance was observed between control and infected samples at 24 hpi (Figure 6). RNA-seq data revealed 533 DEGs in the infected compared to uninfected hBMECs, of which 318 genes were upregulated and 215 genes were downregulated. These transcriptomic differences are clearly shown in the heatmap (Figure 7). The dendrogram on top of the heatmap shows the clustering of the samples based on gene expression similarities at 6-, 24-, and 48-hpi. Of the 533 DEGs, only five genes were differentially regulated over the entire infection time course. Of these, four genes (*CHAC1*, *DDIT4*, *RPL37AP1*, and *MT-CO2*) were upregulated and one gene (*AHNAK2*) was down-regulated (Table S1). ChaC glutathione-specific γ-glutamylcyclotransferase 1 (*CHAC1*) plays a role in regulating the inflammatory process. DNA damage-inducible transcript 4 (*DDIT4*) is activated under various cellular stresses, including DNA damage, hypoxia, oxidative stress, and starvation. *DDIT4* also contributes to the regulation of various cell functions, including proliferation, apoptosis, and differentiation. *RPL37AP1* (ribosomal protein L37a pseudogene 1), encodes ribosomal protein L37a, a component of 60S large ribosomal subunit, which is involved in protein synthesis from messenger RNAs. The mitochondrially encoded cytochrome c oxidase II (*MT-CO2*) is a component of respiratory complex IV.

**Figure 8:**
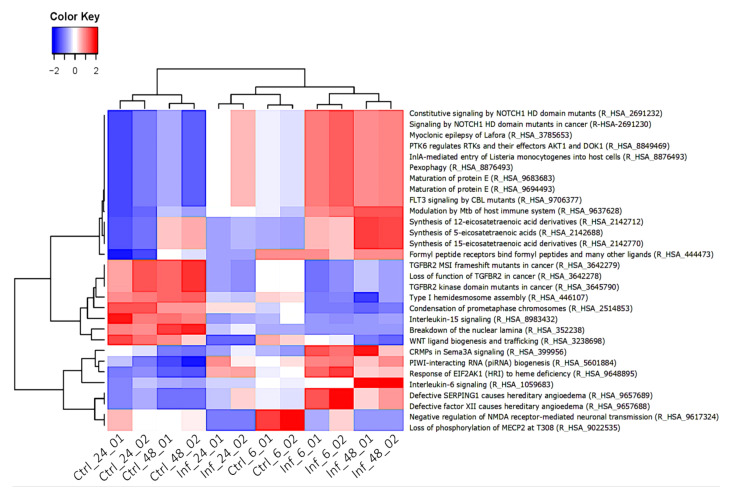
Heatmap clustering representing of the top 30 pathways (FDR < 0.05) in all samples. The color scale from blue (low) to red (high) corresponds to the expression values. The pathway name and Reactome pathway ID are shown on the right side of the heatmap. Columns represent the samples and time points post infection, while rows represent individual pathways. The phylogenetic tree above the heatmap shows the hierarchical clustering of the samples. The relationships between the pathways are shown on the left tree.

Reactome pathway analysis of all DEGs identified 916 significant pathways (FDR < 0.05), 306 of these were downregulated. The top 30 most significantly enriched pathways are shown (Figure 8). The heatmap highlighted interchangeable expression in immune related signaling (i.e., interleukin IL-6 signaling) at different time points after infection. Out of the top 30 most different pathways, 10 pathways were common between 24 and 48 hpi samples, including ADORA2B-mediated anti-inflammatory cytokine production (R_HSA_9660821), CD163 mediating an anti-inflammatory response (R_HSA_9662834), cysteine formation from homocysteine (R_HSA_1614603), interleukin 10 signaling (R_HSA_6783783), interleukin 6 family signaling (R_HSA_6783589), interleukin 6 signaling (R_HSA_1059683), *Leishmania* parasite growth and survival (R_HSA_9664433), MAPK1 (ERK2) activation (R_HSA_112411), MAPK3 (ERK1) activation (R_HSA_110056), and sulfur amino acid metabolism (R_HSA_1614635).

**Figure 9:**
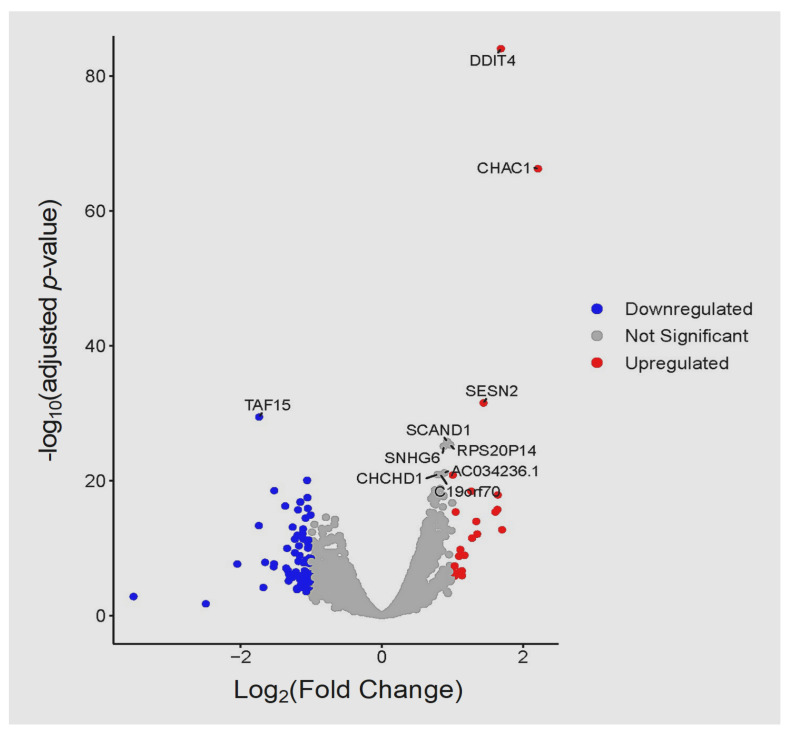
Volcano plot comparing the top 10 DEGs, with the highest differences in expression, between infected and control samples at 6 hpi. Upregulated (red) and downregulated (blue) genes are shown. The fold-changes in gene expression between samples (log_2_FC) are plotted on the *x*-axis and the *y*-axis shows statistical significance of the differences (*p*-values). Each dot in the plot represents one gene.

**Figure 10:**
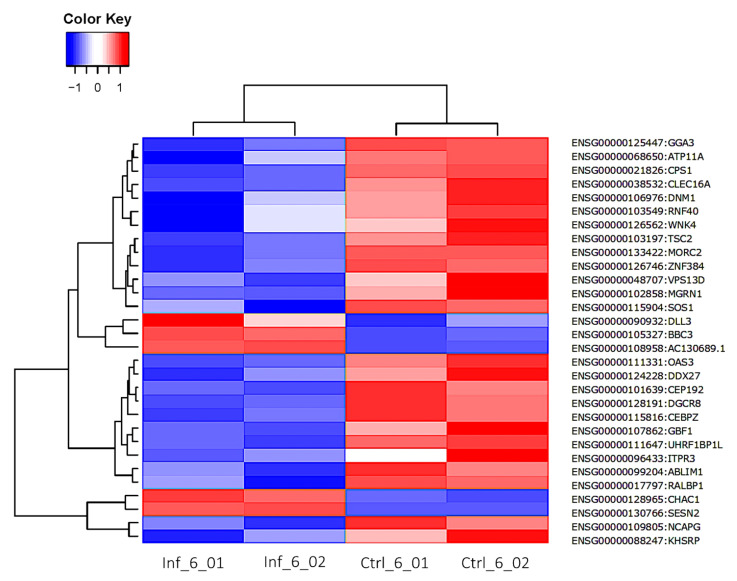
Heatmap generated from the log_2_FC of the top 30 DEGs at 6 hpi. Blue and red colors indicate low and high gene expression, respectively. Gene IDs are indicated on the right side of the heatmap. Columns represent the samples and time points post infection, while rows represent individual genes. The phylogenetic tree above the heatmap shows the hierarchical clustering of the samples. The relationships between the genes are shown on the left tree. For each gene symbol, the respective log_2_FC and other information can be found in Table S2.

At 6 hpi, 97 DEGs were identified, 23 of these were up-regulated and 74 were downregulated (Table S2). The three most up-regulated genes were *CHAC1* (ENSG00000128965, ChaC glutathione specific gamma-glutamylcyclotransferase 1), *PLCB1* (ENSG0000018261, phospholipase C beta 1), and *DDIT4* (ENSG00000168209, DNA damage inducible transcript 4) with log_2_FC of 2.21, 1.70, and 1.69, respectively. The three most down-regulated genes included a novel gene (ENSG00000281181), *RN7SL1* (ENSG00000276168, RNA component of signal recognition particle 7SL1), and *TLN1* (ENSG00000137076, talin 1) with log_2_FC of –3.52, –2.49, and –2.05, respectively. The volcano plot (Figure 9) shows more down-regulated (log_2_FC < −1; FDR < 0.05) than up-regulated (log_2_FC > 1; FDR < 0.05) genes. The heatmap of the top 30 DEGs is shown (Figure 10) with more genes down-regulated than up-regulated in the infected samples. To investigate the biological function of the DEGs induced by *T. gondii* at 6 hpi, the gene list in Table S2 with their expression values from edgeR counting were submitted to the REACTOME database for ssGSEA analysis to derive pathway expression values for every sample. The top 30 most significantly enriched pathways (between control and infected samples) are shown in the heatmap (Figure S7).

**Figure 11:**
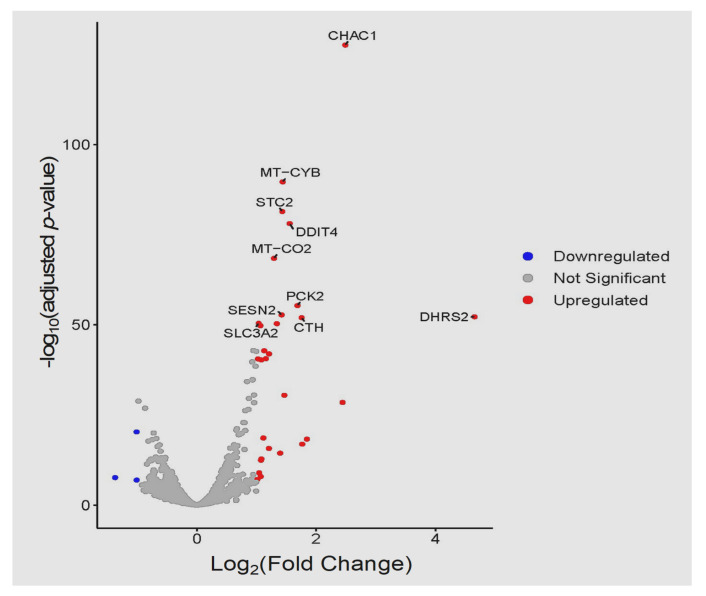
Volcano plot comparing the top 10 DEGs, with the highest differences in expression, between infected and control samples at 24 hpi. Upregulated (red) and downregulated (blue) genes are shown. *p*-values were plotted on the *y*-axis while log_2_FC values are plotted on the *x*-axis. Each dot in the plot represents one gene.

**Figure 12:**
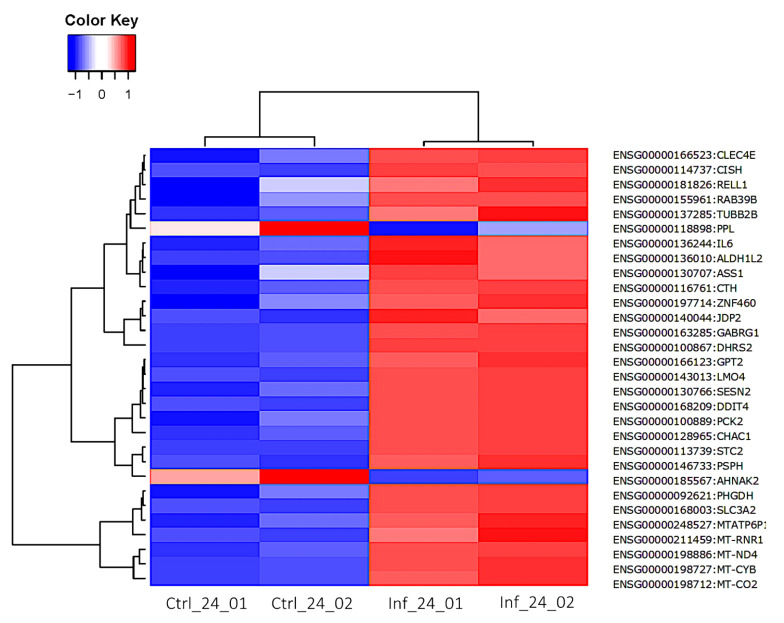
Heatmap generated from the log_2_FC of the top 30 DEGs at 24 hpi. Blue and red colors indicate low and high gene expression, respectively. Gene IDs are indicated on the right side of the heatmap. Columns represent the samples and time points post infection, while rows represent individual genes. The phylogenetic tree above the heatmap demonstrates the hierarchical clustering of the samples. The relationships between the genes are shown on the left tree. For each gene symbol, the respective log2FC and other information can be found in Table S3.

At 24 hpi, 32 DEGs were identified, of these 29 were up-regulated and 3 were down-regulated (Table S3). The three most up-regulated genes included *DHRS2* (ENSG00000100867, dehydrogenase/reductase 2), *CHAC1* (ENSG00000128965, ChaC glutathione specific gamma-glutamylcyclotransferase 1), and *GABRG1* (ENSG00000163285, gamma-aminobutyric acid type A receptor subunit gamma1) with log_2_FC of 4.65, 2.49, and 2.44, respectively. Only three down-regulated genes were detected, including *PPL* (ENSG00000118898, periplakin), *AHNAK2* (ENSG00000185567, AHNAK nucleoprotein 2), and *CCBE1* (ENSG00000183287, collagen and calcium binding EGF domains 1) with log_2_FC –1.37, –1.01, and –1.01, respectively. The volcano plot shows more up-regulated genes than down-regulated genes (Figure 11). The heatmap of the top 30 DEGs is shown (Figure 12) with more genes up-regulated than down-regulated in infected samples. The top 30 most significantly enriched pathways are shown in Figure S8.

**Figure 13:**
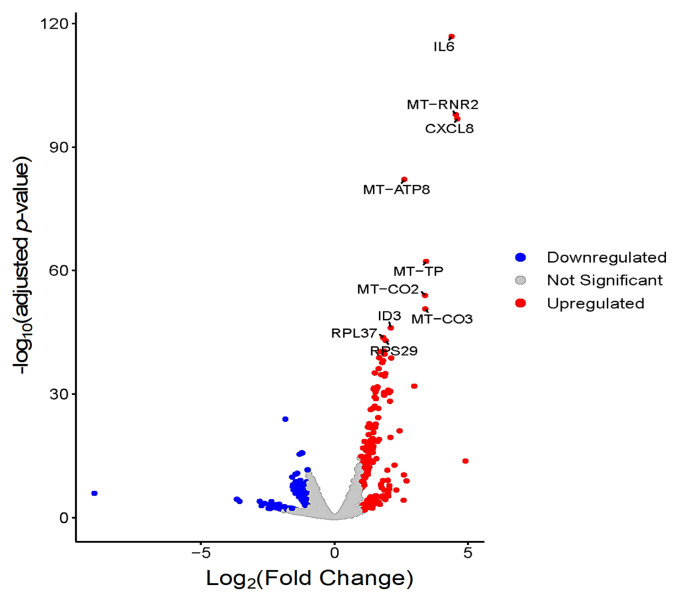
Volcano plot comparing the top 10 genes with the highest differences in expression between infected and control samples at 48 hpi. Upregulated (red) and downregulated (blue) genes are shown. *p*-values were plotted on the *y*-axis while log_2_FC values are plotted on the *x*-axis. Each dot in the plot represents one gene.

**Figure 14:**
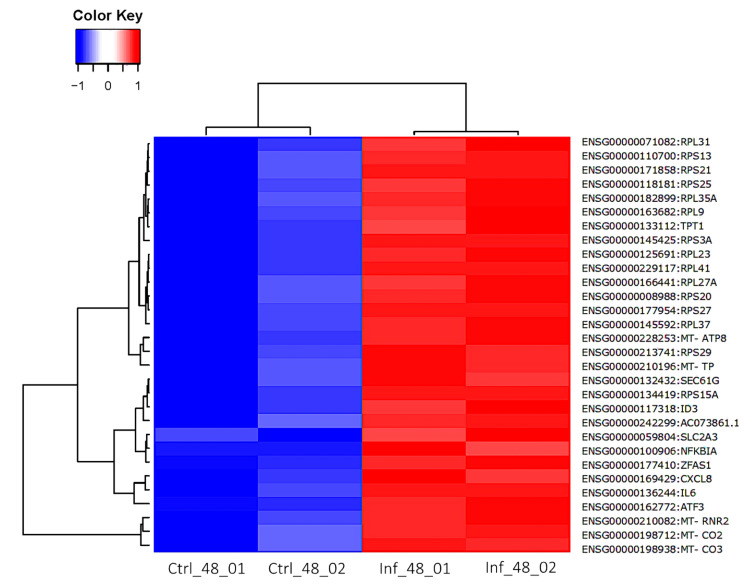
Heatmap generated from the log_2_FC of the top 30 DEGs at 48 hpi. Blue and red colors indicate low and high gene expression, respectively. Gene IDs are indicated on the right side of the heatmap. Columns represent the samples and time points post infection, while rows represent individual genes. The phylogenetic tree above the heatmap demonstrates the hierarchical clustering of the samples. The relationships between the genes are shown on the left tree. For each gene symbol, the respective log_2_ FC and other information can be found in Table S4.

At 48 hpi, 404 DEGs were identified, of these 266 were up-regulated and 138 were down-regulated (Table S4). The three most up-regulated genes were *NR4A3* (ENSG00000119508, nuclear receptor subfamily 4 group A member 3), *CXCL8* (ENSG00000169429, C-X-C motif chemokine ligand 8), and *MT-RNR2* (ENSG00000210082, (mitochondrially encoded 16S rRNA) with log_2_FC of 4.89, 4.59, and 4.54, respectively. *NR4A3* is a member of nuclear receptor subfamily 4, which is an important regulator of cellular function and inflammatory signaling. The chemotactic pro-inflammatory cytokine interleukin-8 (CXCL8) plays a role in host response to intracellular pathogens. The *MT-RNR2* (16S RNA) is a mitochondrial-based gene which encodes one rRNA subunit of mitochondrial ribosomes. The three most down-regulated genes are *TMCC2* (ENSG00000133069, transmembrane and coiled-coil domain family 2), *ZNF101* (ENSG00000181896, zinc finger protein 101), and *LCA5* (ENSG00000135338, lebercilin LCA5), with log_2_FC of –8.99, –3.65, and –3.55, respectively. The volcano plot shows more up-regulated genes than down-regulated genes (Figure 13). The top 30 DEGs are shown in heatmap (Figure 14), where all genes were up-regulated in infected samples. The top 30 most significantly enriched pathways are shown in the heatmap (Figure S9).

## 4. Discussion

Tracking the molecular changes of host cells in response to infection progression is fundamental for the understanding of host-pathogen interaction and the development of therapeutic interventions. Label-free spectroscopic imaging has been emerging as a central tool for elucidating the global chemical changes caused by infection with obligate intracellular protozoan parasites, such as *T. gondii* [4,9]. FTIR spectroscopy is an effective label-free imaging tool that can detect the (bio)molecular constituents of individual host cells without the use of fluorescent probes [27]. In the present study, we investigated the general dynamics of chemical changes that accompany *T. gondii* infection of hBMECs using conventional FTIR micro spectroscopic analysis, which revealed no significant differences between infected and control cells at 24 hpi or 48 hpi in PC1. This result suggests that spectral changes that are not specific to infection were strong despite the rigorous data pretreatment processes applied. This could also be due to lower spectral signal-to-noise ratio when compared to SR-FTIR or artefacts associated with the measurement of dried cells. However, significant differences were observed in PC2 with changes mainly associated with the lipid CH stretching bands and protein amide I and II. This result corroborates a previous study where clustering analysis of the FTIR spectral data showed an alteration in the lipid to protein ratio in the infected cells, which was confirmed by biochemical assays [2]. At 24 hpi, infected cells had an overall lower lipid content, however at 48 hpi, the lipid content was higher in the infected cells.

Although FTIR spectroscopy has been previously used to distinguish FTIR spectra of *T. gondii*-infected cells from those of uninfected cells [2], we argue that there is a need to dissociate the differences between infected and uninfected cells not only at the single cell level, but also at the sub-cellular scale. Therefore, we used synchrotron based FTIR microspectroscopy to investigate whether FTIR spectral data can be used to study *T. gondii* infection-driven chemical alterations at the sub-cellular level. SR-FTIR ZnS hemisphere analysis showed that infected cells had a lower lipid content in the cytosol region of infected cells at 24 hpi. The overall lipid content was however higher in the infected cells at 48 hpi, where PCA of all three subcellular regions with the most distinct differences (i.e., lipid-rich cytosol, nucleus, or lipid-poor cytosol) showed a higher lipid content. In the lipid poor region of the cytosol, PC1 has shown a significant difference between control and infected cells with changes mainly found in the protein amide I and II peaks. These changes are consistent with the findings obtained by conventional FTIR analysis.

Alterations in the lipid and protein contents of hBMECs have been previously detected using proton nuclear magnetic resonance-based metabolomic analysis [7,9]. In addition, changes in the abundance of lipids (e.g., glycerophospholipid) and amino acid (phenylalanine, retinol, and tryptophan) were detected in mouse cerebellum during *T. gondii* infection by using ultra performance liquid chromatography-tandem mass spectrometry-based metabolomics [28]. However, the application of these methods requires a special sample preparation, which may alter the biochemical composition of the host cells. In contrast, vibrational spectroscopy techniques, such as the FTIR used in the present study, can be applied in nondestructive and label-free analysis of biochemical changes in the host cells. The chemical changes in infected host cells can be significant enough to be inferred from the FTIR spectra at the single cell level, whereas SR-FTIR ZnS hemisphere analysis can provide insight into changes of the chemical composition of host cells at the sub-cellular level. The application of conventional FTIR and synchrotron FTIR clearly showed how these methods can detect infection-related molecular changes as early as 24 hpi. Importantly, these approaches can be applied for live-cell imaging, increasing the scope for further investigation of host cell-parasite dynamics. It is noteworthy to mention that conventional micro FTIR was used for imaging whole cells while SR-based FTIR offered subcellular spatial resolution, which complicates direct quantitative comparisons of spectra obtained by the two imaging approaches.

RNA-seq, a high-throughput method for characterizing transcriptomes, was used to compare the transcription levels of genes between *T. gondii*-infected and control hBMECs at 6, 24 and 48 hpi. We hypothesized that global profiling of transcriptional changes in *T. gondii*-infected versus uninfected cells could elucidate the metabolic and regulatory mechanisms involved in host cell response to *T. gondii* infection. *T. gondii* infection altered the expression of genes involved in cellular processes and regulatory events, such as immune signaling, adaptation to stress, and amino acid metabolism. Sulphur amino acid metabolism was one of the pathways commonly found between 24 and 48 hpi samples. This pathway can be modulated by *T. gondii* protein (elongation factor 1-alpha, putative; (EF-1-alpha). Immunization of mice using EF-1-alpha-based monoclonal antibody vaccine increased survival of mice against *T. gondii* infection, suggesting the immunogenicity of EF-1-alpha and its involvement in mediating host-*T. gondii* interaction in mice [29]. What roles other *T. gondii* effector proteins play in the modulation of the host sulfur amino acid metabolism remain to be elucidated.

Pathway analysis also revealed that transcriptomic changes were predominantly part of a defense mechanism involving key cytokines (e.g., IL-6, IL-8, IL-10). IL-6 and IL-8 are involved in the development of protective immunity against *T. gondii*, whereas IL-10 is required to limit *T. gondii* infection-induced pathology. This immune-balance could be an advantage for *T. gondii* if a large proportion of host cell gene expression is directed at immune responses, and *T. gondii* already possesses mechanisms for countering these. Other host processes may then be easier to interfere with and hijacked by *T. gondii*. The results of the present and previous studies [30,31,32,33,34,35], confirm the ability of *T. gondii* to manipulate various cellular processes, particularly immune response via reprogramming host gene expression to support its own survival within the host cells.

## 5. Conclusions

We have investigated the molecular mechanisms underlying the interactions of *T. gondii* with its host cell at the biochemical and transcriptomic level, which is the first characterization of the dynamics of *T. gondii* infection at this level of detail. We applied conventional and synchrotron based FTIR microspectroscopy to elucidate the chemical changes that are associated with *T. gondii* infection of hBMECs. We studied how the lipid, protein, and nucleic acid compositions change after exposure to infection using information from the FTIR spectra. Our results showed that FTIR spectroscopy delivers the necessary resolution to uncover chemical alterations within individual cells and at the sub-cellular level. Conventional FTIR analysis and SR-FTIR ZnS hemisphere approach showed that the lipid content of infected cells was decreased at 24 hpi but increased at 48 hpi. These label free imaging methods improved our knowledge of the cellular processes taking place during *T. gondii* infection. Transcriptomic analysis revealed genes and signaling pathways that play various roles in mediating the host cell response to *T. gondii* infection. To determine the involvement of specific molecular species will require the integration of these chemical imaging tools with targeted analytical approaches, such as isotope labeling methods and substrate enrichments, and the further investigation of how the host cells respond and adapt to *T. gondii* infection.

## Data Availability

The RNA sequencing reads have been deposited in the ArrayExpress of the European Bioinformatics Institute under accession no. E-MTAB-11515.

## Supplementary Materials

The following are available online at https://www.mdpi.com/article/10.3390/cells11050811/s1, Figure S1: FTIR images (integration range shown on the left column) of the three other host cells at 24 hpi. Scale bars = 10 μm, Figure S2: FTIR images (integration range shown on the left column) of the three other control cells at 24 h. The visible images were not collected for two cells. Scale bars = 10 μm, Figure S3: FTIR images (integration range shown on the left column) of the three other cells at 48 hpi. Scale bars = 10 μm, Figure S4: FTIR images (integration range shown on the left column) of the three other control cells at 48 h. Scale bars = 10 μm, Figure S5: The extracted spectra of infected and control cells at 24 hpi from the lipid-rich, DNA-rich, and lipid-poor regions, Figure S6: The extracted spectra of infected and control cells at 48 hpi from the lipid-rich, DNA-rich, and lipid-poor regions, Figure S7: The top 30 pathways most significantly activated (red) or inactivated (blue) at 6 hpi by *T. gondii*. Columns represent replicates of the samples and rows represent individual pathways. The phylogenetic tree above the heatmap demonstrates the hierarchical clustering of the samples. The relationships between the pathways are shown on the left tree, Figure S8: The top 30 pathways most significantly activated (red) or inactivated (blue) at 24 hpi by *T. gondii*. Columns represent replicates of the samples and rows represent individual pathways. The phylogenetic tree above the heatmap demonstrates the hierarchical clustering of the samples. The relationships between the pathways are shown on the left tree, Figure S9: The top 30 pathways most significantly activated (red) or inactivated (blue) at 48 hpi by *T. gondii*. Columns represent replicates of the samples and rows represent individual pathways. The phylogenetic tree above the heatmap demonstrates the hierarchical clustering of the samples. The relationships between the pathways are shown on the left tree, Table S1: Differentially expressed genes commonly detected at 6-, 24-, and 48-hpi by *T. gondii*, Table S2: List of the 97 differentially expressed genes (23 upregulated and 74 downregulated) with FDR < 0.05 and absolute log_2_FC < −1 and > 1 at 6 hpi. The respective gene identifier, gene symbol, and log_2_FC are shown, Table S3: List of the 32 differentially expressed genes (29 up-regulated and 3 down-regulated) with FDR < 0.05 and absolute log_2_FC < −1 and > 1 at 24 hpi. The respective gene identifier, gene symbol, and log_2_ FC are shown, Table S4: List of the 404 differentially expressed genes (266 up-regulated and 138 down-regulated) with FDR < 0.05 and absolute log_2_FC < −1 and > 1 at 48 hpi. The respective gene identifier, gene symbol, and log_2_FC are shown.
