# Supplementary material for: Illuminating Host-Parasite Interaction at the Cellular and Subcellular Levels with Infrared Microspectroscopy
Source: Cells. 2022 Feb 25;11(5):811. doi: 10.3390/cells11050811 (PMC8909495; doi:10.3390/cells11050811)
Supplement: Supplementary file 1 [file cells-11-00811-s001.zip › cells-1547901 -Supplementary materials.pdf]

Supplementary materials:

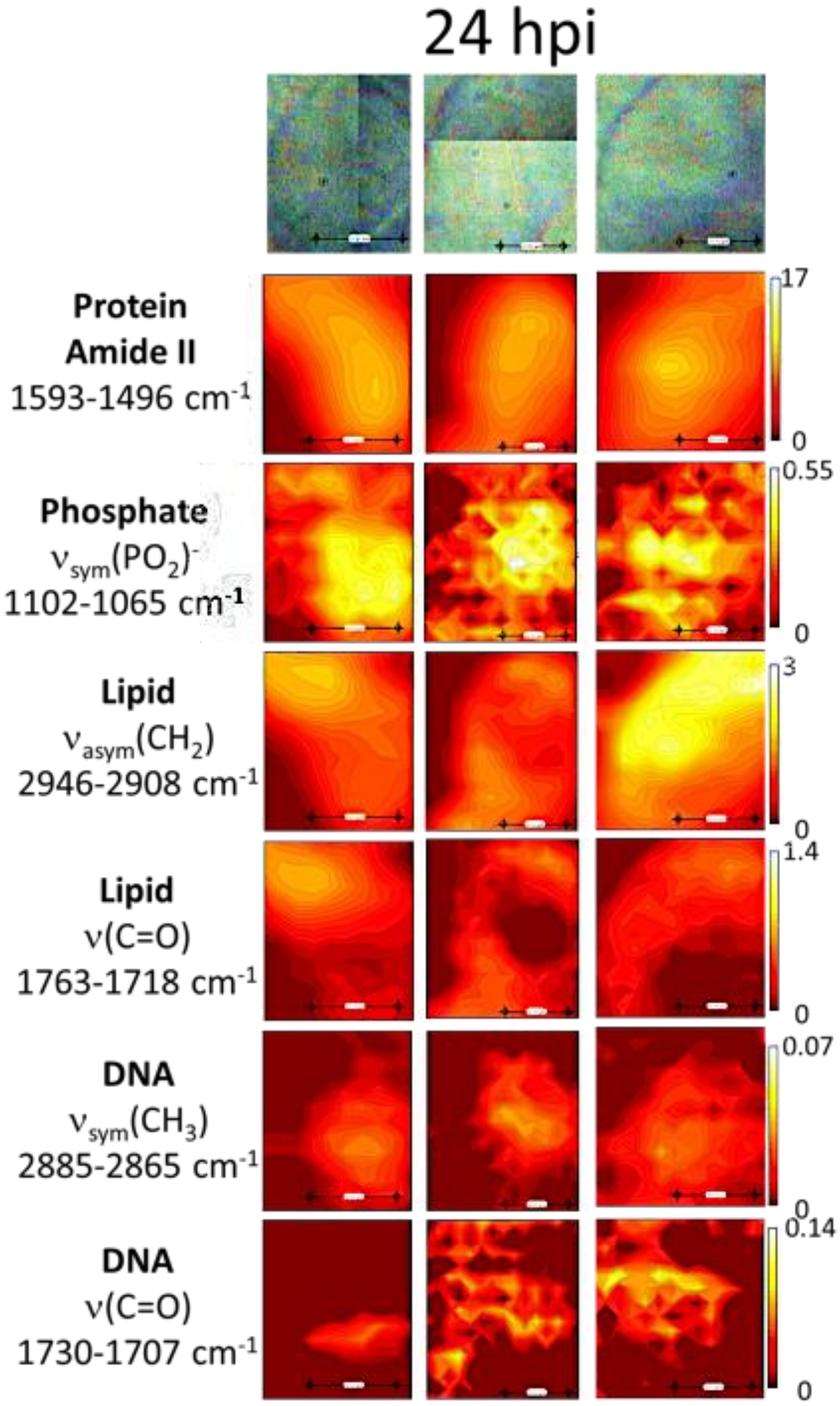

Figure S1: FTIR images (integration range shown on the left column) of the three other host cells at 24 hpi. Scale bars = 10 μm.

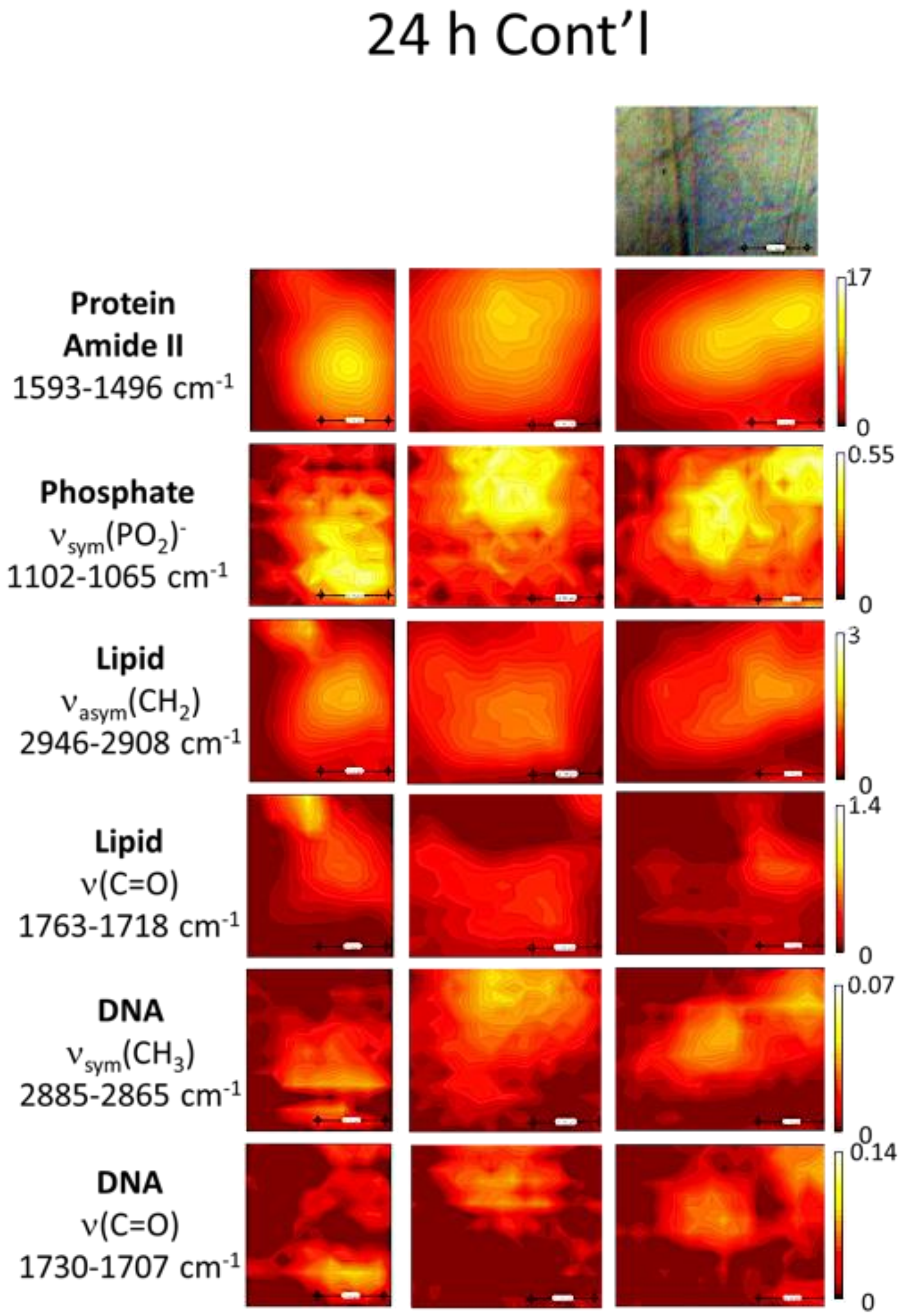

Figure S2: FTIR images (integration range shown on the left column) of the three other control cells at 24 h. The visible images were not collected for two cells. Scale bars = 10 μm.

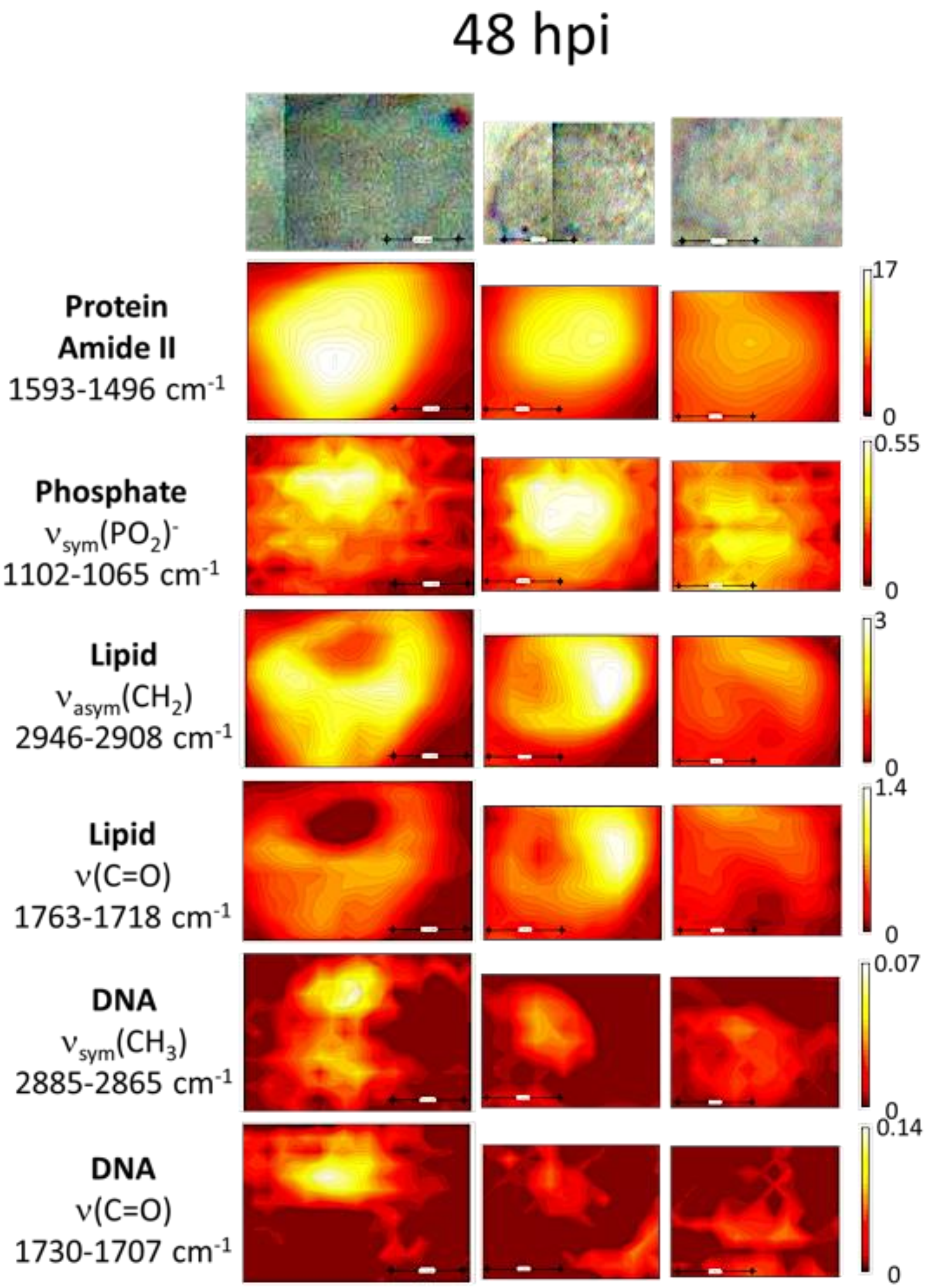

Figure S3: FTIR images (integration range shown on the left column) of the three other cells at 48 hpi. Scale bars = 10 μm.

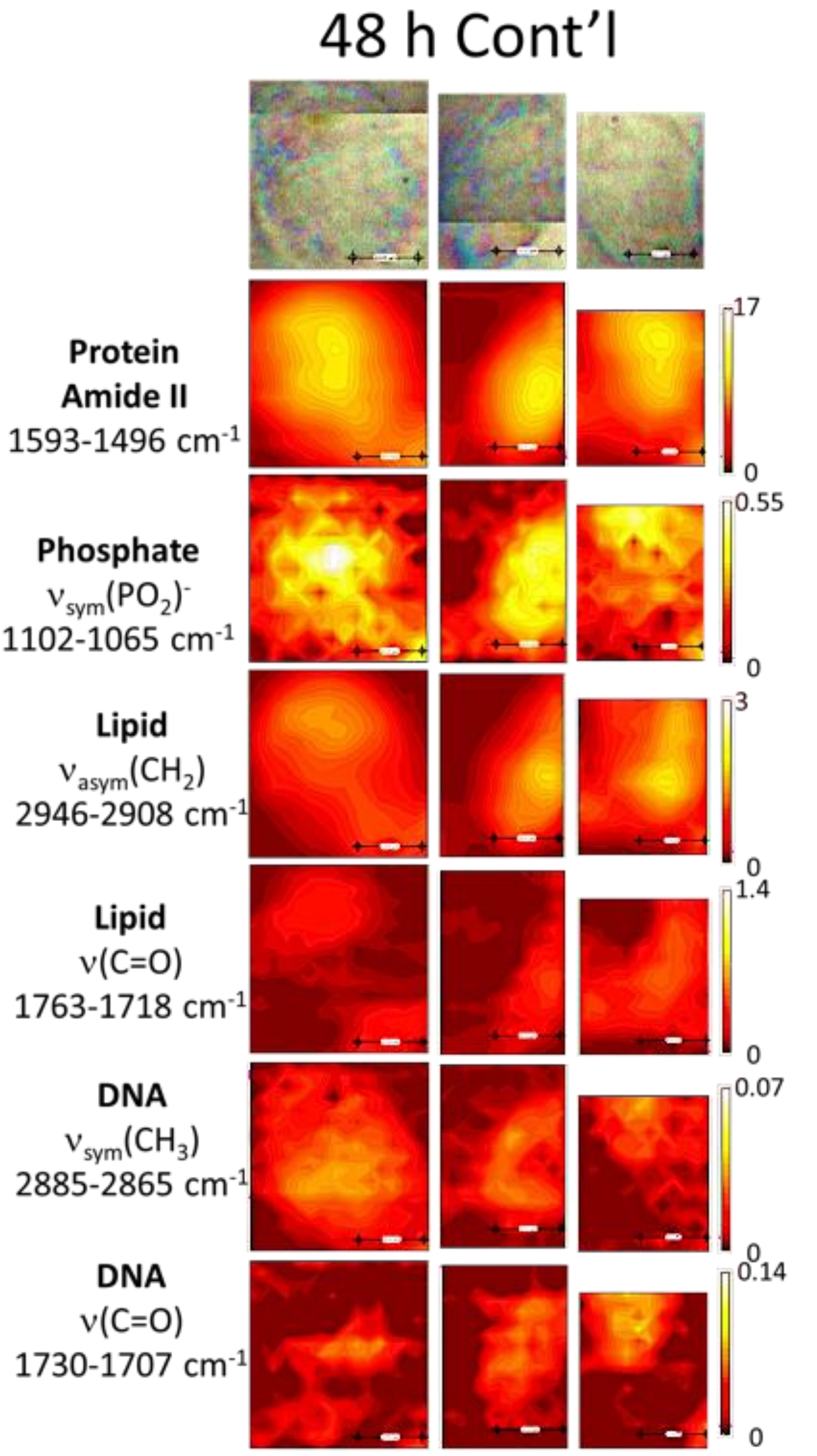

Figure S4: FTIR images (integration range shown on the left column) of the three other control cells at 48 h. Scale bars = 10 μm.

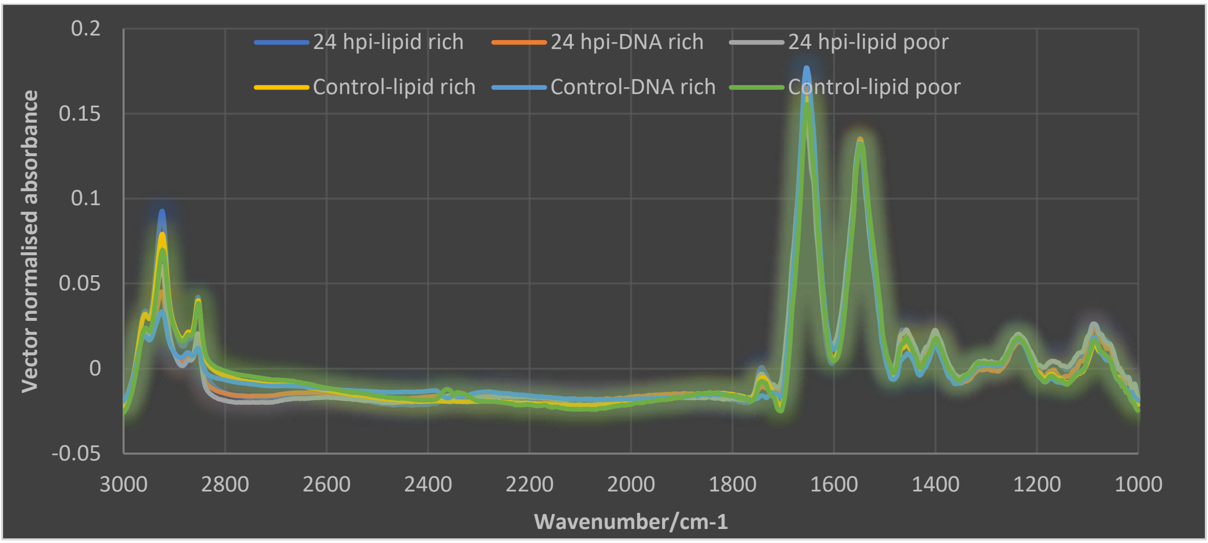

Figure S5: The extracted spectra of infected and control cells at 24 hpi from the lipid-rich, DNA-rich, and lipid-poor regions.

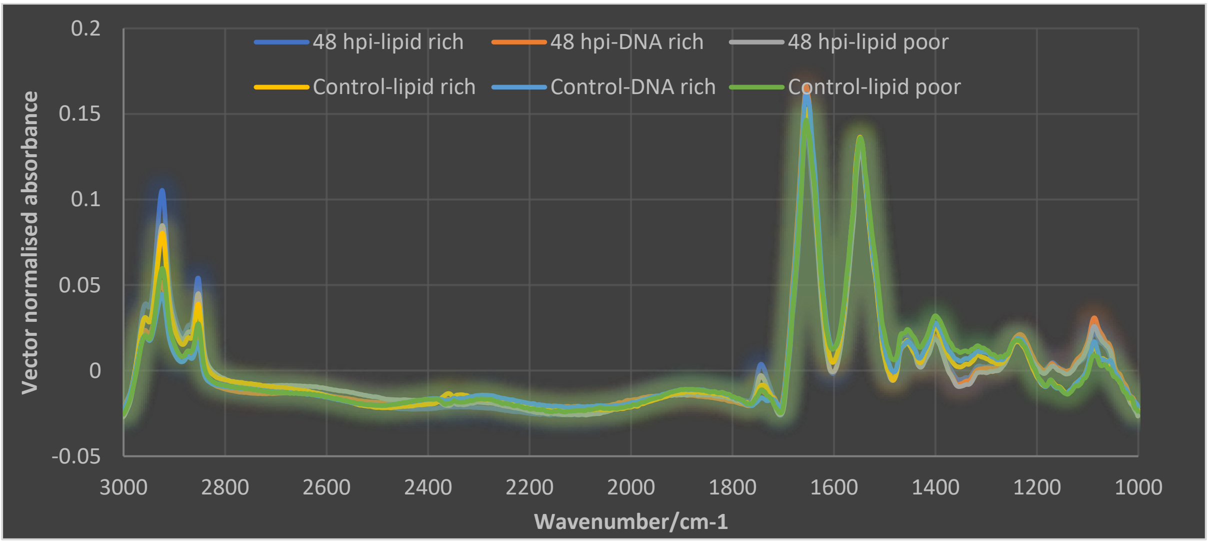

Figure S6: The extracted spectra of infected and control cells at 48 hpi from the lipid-rich, DNA-rich, and lipid-poor regions.

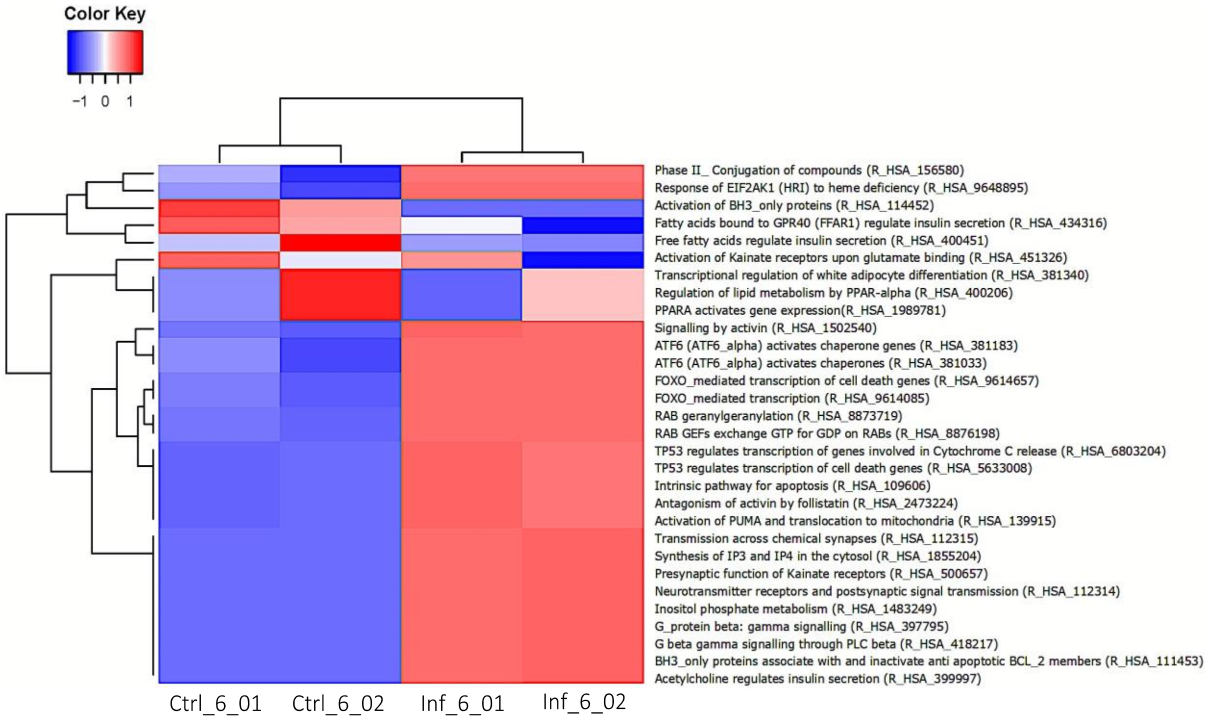

Figure S7. The top 30 pathways most significantly activated (red) or inactivated (blue) at 6 hpi by *T. gondii*. Columns represent replicates of the samples and rows represent individual pathways. The phylogenetic tree above the heatmap demonstrates the hierarchical clustering of the samples. The relationships between the pathways are shown on the left tree.

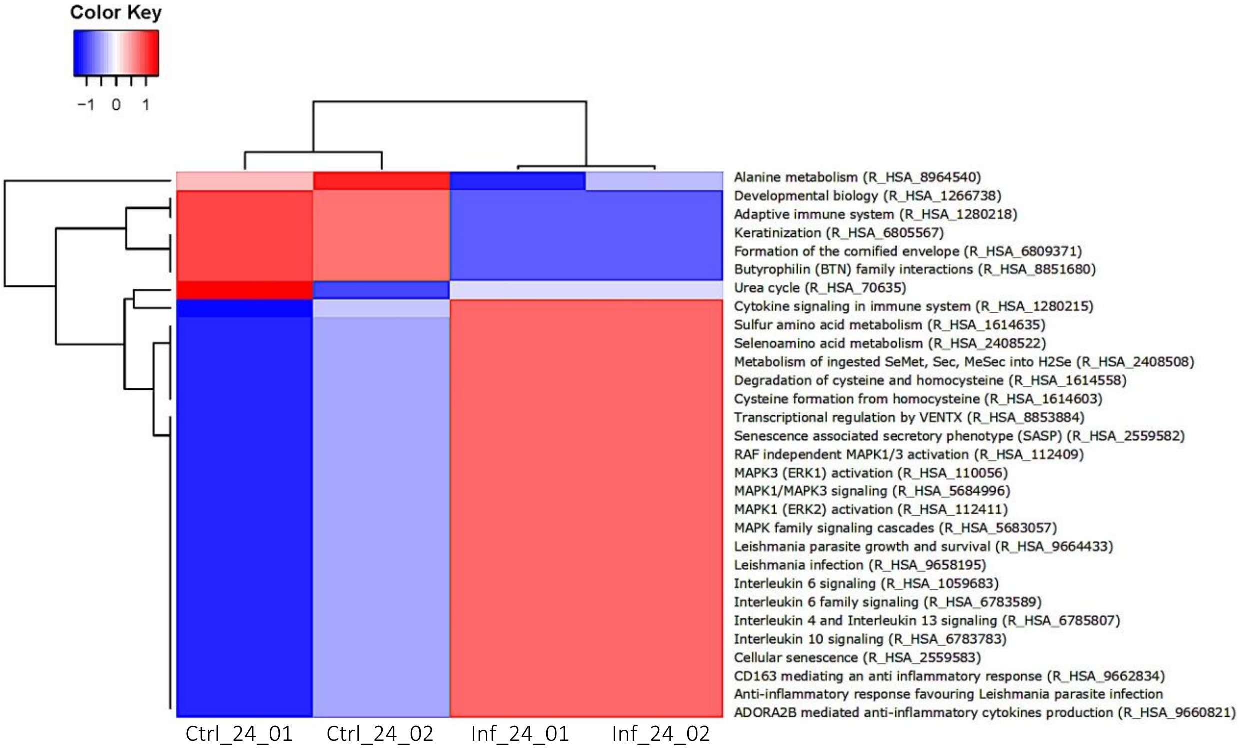

Figure S8. The top 30 pathways most significantly activated (red) or inactivated (blue) at 24 hpi by *T. gondii*. Columns represent replicates of the samples and rows represent individual pathways. The phylogenetic tree above the heatmap demonstrates the hierarchical clustering of the samples. The relationships between the pathways are shown on the left tree.

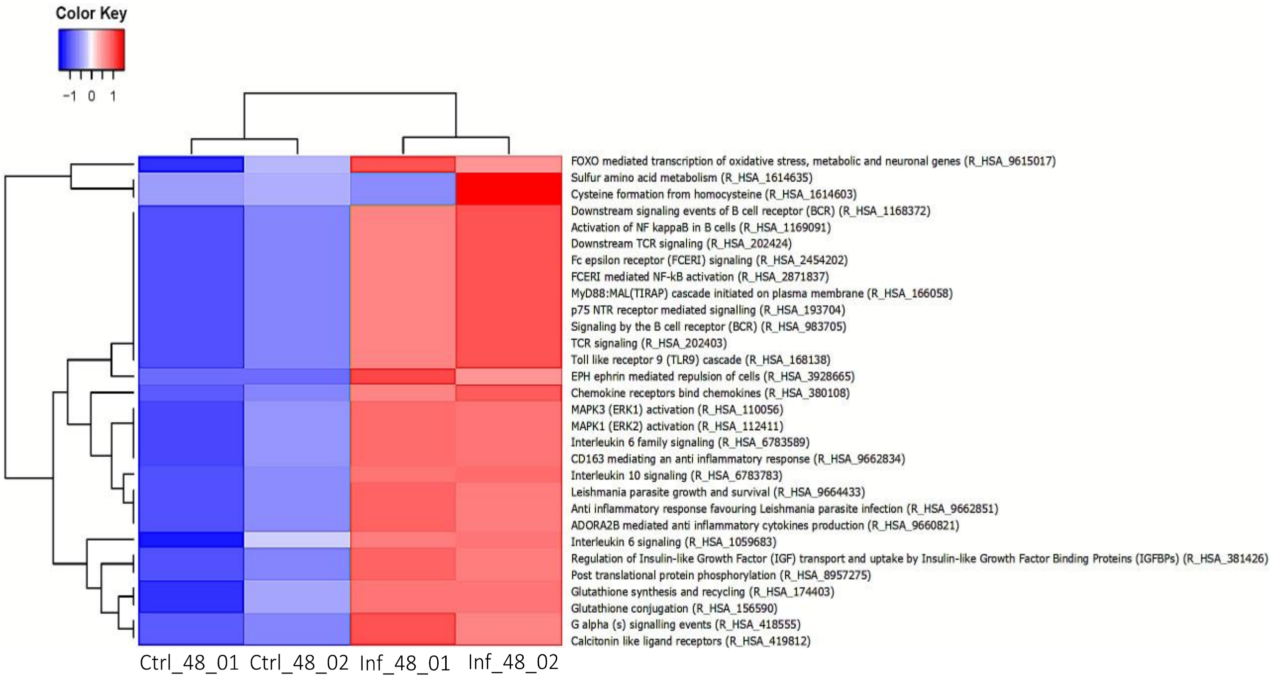

Figure S9. The top 30 pathways most significantly activated (red) or inactivated (blue) at 48 hpi by *T. gondii*. Columns represent replicates of the samples and rows represent individual pathways. The phylogenetic tree above the heatmap demonstrates the hierarchical clustering of the samples. The relationships between the pathways are shown on the left tree.

Table S1. Differentially expressed genes commonly detected at 6-, 24-, and 48-hours post *Toxoplasma gondii* infection.

No	Gene_ID	Gene Symbol	Log_2 FC		
			6 hpi	24 hpi	48 hpi
1	ENSG00000128965	CHAC1	2.21	2.49	2.44
2	ENSG00000168209	DDIT4	1.69	1.56	2.05
3	ENSG00000226243	RPL37AP1	1.11	1.01	1.87
4	ENSG00000198712	MT-CO2	1.35	1.29	3.39
5	ENSG00000185567	AHNAK2	-1.53	-1.01	-1.84

Table S2. List of the 97 differentially expressed genes (23 upregulated and 74 downregulated) with FDR < 0.05 and absolute log_2 FC < -1 and > 1 at 6 hpi. The respective gene identifier, gene symbol, and log_2 FC are shown.

No	Gene_ID	Gene Symbol	Log_2 FC	logCPM	FDR
1	ENSG00000128965	CHAC1	2.21137	5.699	1.15E-63
2	ENSG00000182621	PLCB1	1.7036	2.0725	9.68E-12
3	ENSG00000168209	DDIT4	1.6865	6.0611	3.60E-81
4	ENSG00000198938	MT-CO3	1.64226	15.374	2.88E-16
5	ENSG00000198840	MT-ND3	1.63625	12.4749	2.27E-14
6	ENSG00000198727	MT-CYB	1.60798	12.4027	5.04E-14
7	ENSG00000130766	SESN2	1.43969	5.4179	4.21E-29
8	ENSG00000198712	MT-CO2	1.35035	13.3135	3.70E-11
9	ENSG00000175197	DDIT3	1.33766	3.0946	8.65E-13
10	ENSG00000155961	RAB39B	1.2788	2.818	1.22E-10
11	ENSG00000227063	RPL41P1	1.26597	5.8127	9.92E-17
12	ENSG00000134363	FST	1.17069	3.2283	2.06E-08
13	ENSG00000090932	DLL3	1.13526	3.2414	8.75E-06
14	ENSG00000198786	MT-ND5	1.13293	9.4449	2.17E-06
15	ENSG00000212907	MT-ND4L	1.12138	9.0146	2.90E-06
16	ENSG00000226243	RPL37AP1	1.11171	3.084	3.86E-09
17	ENSG00000232888	RPS11P5	1.09897	2.3982	2.90E-06
18	ENSG00000105327	BBC3	1.09268	2.7863	2.67E-08
19	ENSG00000177600	RPLP2	1.04567	10.2259	5.04E-14
20	ENSG00000214756	CSKMT	1.03111	2.6142	4.81E-07
21	ENSG00000108958	AC130689.1	1.02988	2.1807	1.02E-05
22	ENSG00000164136	IL15	1.02138	2.4457	4.01E-06
23	ENSG00000143013	LMO4	1.00543	5.756	5.62E-19
24	ENSG00000197386	HTT	-1.00121	3.7237	1.34E-07
25	ENSG00000198742	SMURF1	-1.00211	3.464	5.18E-08
26	ENSG00000197299	BLM	-1.00267	2.3634	1.12E-05
27	ENSG00000103197	TSC2	-1.00632	2.5565	5.52E-06
28	ENSG00000138780	GSTCD	-1.00838	3.4833	1.26E-07
29	ENSG00000140525	FANCI	-1.009	5.3883	1.23E-13
30	ENSG00000107862	GBF1	-1.00904	3.8097	4.08E-08
31	ENSG00000174373	RALGAPA1	-1.01566	1.9359	6.12E-05
32	ENSG00000048707	VPS13D	-1.01832	2.4049	7.56E-05
33	ENSG00000088247	KHSRP	-1.02324	5.3564	4.40E-08
34	ENSG00000157933	SKI	-1.0237	3.4035	7.09E-08
35	ENSG00000130702	LAMA5	-1.02883	3.9008	3.06E-04
36	ENSG00000038532	CLEC16A	-1.03078	2.1045	4.32E-05
37	ENSG00000124228	DDX27	-1.03166	3.4793	2.29E-07
38	ENSG00000144583	MARCH4	-1.03693	3.782	2.08E-10

39	ENSG00000184661	CDCA2	-1.03904	3.6099	1.25E-09
40	ENSG00000099204	ABLIM1	-1.04389	4.0519	5.13E-10
41	ENSG00000177084	POLE	-1.04659	3.6927	2.55E-09
42	ENSG00000166326	TRIM44	-1.0482	5.2116	1.56E-14
43	ENSG00000133816	MICAL2	-1.0532	6.8148	5.68E-16
44	ENSG00000126243	LRFN3	-1.05377	1.8531	1.04E-04
45	ENSG00000173692	PSMD1	-1.05693	5.6717	3.09E-18
46	ENSG00000135048	TMEM2	-1.06374	2.3927	3.95E-06
47	ENSG00000181222	POLR2A	-1.0693	4.0283	1.12E-03
48	ENSG00000241973	PI4KA	-1.072	3.3148	8.59E-08
49	ENSG00000126746	ZNF384	-1.07243	2.3625	3.09E-06
50	ENSG00000068650	ATP11A	-1.07535	1.9392	8.29E-05
51	ENSG00000109805	NCAPG	-1.07933	5.3892	3.28E-13
52	ENSG00000163913	IFT122	-1.08473	1.9409	3.65E-05
53	ENSG00000125447	GGA3	-1.08958	1.9578	1.92E-05
54	ENSG00000189057	FAM111B	-1.09305	2.432	2.19E-06
55	ENSG00000151422	FER	-1.10157	2.0018	2.99E-05
56	ENSG00000133422	MORC2	-1.10525	2.5594	1.87E-07
57	ENSG00000017797	RALBP1	-1.11206	3.9272	1.51E-10
58	ENSG00000160294	MCM3AP	-1.11556	4.025	7.95E-12
59	ENSG00000102858	MGRN1	-1.12002	2.2899	9.79E-06
60	ENSG00000111647	UHRF1BP1L	-1.12193	3.6976	3.47E-11
61	ENSG00000135913	USP37	-1.12271	1.9356	1.66E-05
62	ENSG00000172037	LAMB2	-1.14375	2.252	3.19E-04
63	ENSG00000126461	SCAF1	-1.15202	3.9427	1.83E-04
64	ENSG00000253729	PRKDC	-1.15397	5.5036	2.28E-15
65	ENSG00000106976	DNM1	-1.15565	2.1424	3.15E-05
66	ENSG00000021826	CPS1	-1.15789	1.846	1.01E-05
67	ENSG00000179134	SAMD4B	-1.16254	3.5256	2.25E-08
68	ENSG00000128191	DGCR8	-1.17314	3.3565	1.20E-09
69	ENSG00000115904	SOS1	-1.1808	2.6081	1.24E-07
70	ENSG00000138385	SSB	-1.18608	4.0713	2.50E-14
71	ENSG00000070047	PHRF1	-1.19024	2.2739	4.86E-04
72	ENSG00000111331	OAS3	-1.19377	3.5845	5.68E-11
73	ENSG00000122515	ZMIZ2	-1.19984	3.3129	4.26E-04
74	ENSG00000100345	MYH9	-1.20069	6.9686	6.06E-04
75	ENSG00000196547	MAN2A2	-1.21683	1.9157	3.14E-06
76	ENSG00000136731	UGGT1	-1.22896	3.4022	1.03E-08
77	ENSG00000101639	CEP192	-1.22989	3.3853	1.59E-10
78	ENSG00000134697	GNL2	-1.26171	3.7116	4.42E-12
79	ENSG00000096433	ITPR3	-1.265	3.7242	1.53E-05
80	ENSG00000178209	PLEC	-1.27706	6.4746	7.26E-06

81	ENSG00000197694	SPTAN1	-1.30293	4.1234	1.67E-05
82	ENSG00000198420	TCAF1	-1.31775	2.1163	5.15E-06
83	ENSG00000126562	WNK4	-1.31967	2.074	4.48E-05
84	ENSG00000177303	CASKIN2	-1.32918	2.0119	1.97E-06
85	ENSG00000169258	GPRIN1	-1.34077	2.5535	2.68E-09
86	ENSG00000161847	RAVER1	-1.35497	2.1091	9.64E-07
87	ENSG00000171824	EXOSC10	-1.36715	3.6313	7.30E-15
88	ENSG00000115816	CEBPZ	-1.52237	3.6478	8.21E-17
89	ENSG00000185567	AHNAK2	-1.52662	4.1079	2.61E-07
90	ENSG00000196924	FLNA	-1.5298	7.9027	6.31E-07
91	ENSG00000103549	RNF40	-1.65268	2.2308	1.62E-07
92	ENSG00000184634	MED12	-1.67725	2.0901	3.35E-04
93	ENSG00000270647	TAF15	-1.73698	4.3072	3.82E-27
94	ENSG00000166477	LEO1	-1.74083	2.2756	2.85E-12
95	ENSG00000137076	TLN1	-2.04626	4.7947	2.71E-07
96	ENSG00000276168	RN7SL1	-2.49191	7.202	4.12E-02
97	ENSG00000281181	Novel transcript*	-3.51519	4.279	4.84E-03

* **S**imilar to YY1 associated myogenesis RNA 1 (YAM1)

Table S3. List of the 32 differentially expressed genes (29 up-regulated and 3 down-regulated) with FDR < 0.05 and absolute $\log_2$ FC < -1 and > 1 at 24 hpi. The respective gene identifier, gene symbol, and $\log_2$ FC are shown.

No	Gene_ID	Gene Symbol	Log_2 FC	logCPM	FDR
1	ENSG00000100867	DHRS2	4.65183	1.7045	3.08E-50
2	ENSG00000128965	CHAC1	2.48657	4.9748	1.10E-124
3	ENSG00000163285	GABRG1	2.44108	1.9422	4.69E-27
4	ENSG00000197714	ZNF460	1.84396	2.1093	4.17E-17
5	ENSG00000140044	JDP2	1.76444	2.0172	1.01E-15
6	ENSG00000116761	CTH	1.75532	3.9866	4.75E-50
7	ENSG00000100889	PCK2	1.68594	5.9132	3.21E-53
8	ENSG00000168209	DDIT4	1.55512	5.6838	8.35E-76
9	ENSG00000136244	IL6	1.46558	3.7312	5.53E-29
10	ENSG00000198727	MT-CYB	1.43822	12.1219	4.88E-87
11	ENSG00000113739	STC2	1.43145	6.9975	5.17E-79
12	ENSG00000130766	SESN2	1.4203	5.451	1.02E-50
13	ENSG00000155961	RAB39B	1.39579	2.7984	2.59E-13
14	ENSG00000092621	PHGDH	1.34029	8.8939	1.73E-48
15	ENSG00000198712	MT-CO2	1.29304	13.2035	2.90E-66
16	ENSG00000211459	MT-RNR1	1.20914	10.0489	2.87E-40
17	ENSG00000166523	CLEC4E	1.20752	3.1674	1.36E-14
18	ENSG00000166123	GPT2	1.15621	5.5721	5.87E-39
19	ENSG00000248527	MTATP6P1	1.1301	9.1762	4.40E-41
20	ENSG00000136010	ALDH1L2	1.1149	3.6345	2.26E-17
21	ENSG00000114737	CISH	1.08174	3.1206	8.14E-12
22	ENSG00000143013	LMO4	1.07914	5.5082	1.19E-38
23	ENSG00000130707	ASS1	1.07563	3.5248	1.68E-11
24	ENSG00000137285	TUBB2B	1.07022	2.2824	2.32E-07
25	ENSG00000198886	MT-ND4	1.06488	12.5677	6.24E-48
26	ENSG00000181826	RELL1	1.04415	3.1952	2.54E-08
27	ENSG00000168003	SLC3A2	1.03673	8.8154	1.57E-48
28	ENSG00000146733	PSPH	1.02513	6.7976	6.31E-39
29	ENSG00000226243	RPL37AP1	1.01279	2.5033	1.24E-06
30	ENSG00000183287	CCBE1	-1.00718	2.2271	1.82E-06
31	ENSG00000185567	AHNAK2	-1.00728	6.9849	4.92E-19
32	ENSG00000118898	PPL	-1.36762	3.1399	4.11E-07

Table S4. List of the 404 differentially expressed genes (266 up-regulated and 138 down-regulated) with FDR < 0.05 and absolute log_2 FC < -1 and > 1 at 48 hpi. The respective gene identifier, gene symbol, and log_2 FC are shown.

No	Gene_ID	Gene Symbol	Log_2 FC	logCPM	FDR
1	ENSG00000119508	NR4A3	4.89913	-0.33582	8.65E-13
2	ENSG00000169429	CXCL8	4.59705	5.34506	1.92E-94
3	ENSG00000210082	MT-RNR2	4.53753	16.12291	2.57E-95
4	ENSG00000136244	IL6	4.38098	4.66175	6.07E-114
5	ENSG00000210196	MT-TP	3.42679	9.33521	5.04E-60
6	ENSG00000198938	MT-CO3	3.39538	16.17062	1.31E-48
7	ENSG00000198712	MT-CO2	3.38562	15.51201	8.21E-52
8	ENSG00000162772	ATF3	2.98378	3.03837	2.44E-30
9	ENSG00000247627	MTND4P12	2.69148	1.28943	3.15E-08
10	ENSG00000228253	MT-ATP8	2.6104	9.64426	7.98E-80
11	ENSG00000163661	PTX3	2.59511	1.61938	1.34E-09
12	ENSG00000229344	MTCO2P12	2.58733	1.19358	4.97E-04
13	ENSG00000128965	CHAC1	2.43574	3.22514	7.63E-20
14	ENSG00000173227	SYT12	2.31066	1.58128	3.11E-06
15	ENSG00000148926	ADM	2.24294	2.93426	8.18E-12
16	ENSG00000242299	AC073861.1	2.12022	6.41751	5.62E-37
17	ENSG00000117318	ID3	2.10306	7.02857	4.65E-44
18	ENSG00000198804	MT-CO1	2.0901	15.76861	2.83E-18
19	ENSG00000059804	SLC2A3	2.08677	5.59203	3.26E-29
20	ENSG00000169242	EFNA1	2.07474	5.71657	7.27E-27
21	ENSG00000146678	IGFBP1	2.05227	2.44498	3.66E-07
22	ENSG00000168209	DDIT4	2.05143	5.16872	6.45E-29
23	ENSG00000205763	RP9P	2.04257	1.87286	1.90E-06
24	ENSG00000123496	IL13RA2	2.01459	2.23936	6.44E-05
25	ENSG00000100906	NFKBIA	2.00889	5.5154	1.84E-29
26	ENSG00000073756	PTGS2	2.00233	1.76798	1.13E-05
27	ENSG00000276168	RN7SL1	1.99372	2.55734	2.56E-08
28	ENSG00000136826	KLF4	1.98868	2.4373	2.26E-08
29	ENSG00000107984	DKK1	1.97681	3.04423	1.15E-10
30	ENSG00000213741	RPS29	1.91322	9.02634	4.59E-41
31	ENSG00000177410	ZFAS1	1.90158	5.84273	2.47E-33
32	ENSG00000234335	RPS4XP11	1.89987	1.36023	3.56E-04
33	ENSG00000226243	RPL37AP1	1.8719	2.1734	3.66E-06
34	ENSG00000171858	RPS21	1.87049	10.04098	7.45E-38
35	ENSG00000170634	ACYP2	1.86873	1.82501	5.80E-05
36	ENSG00000132432	SEC61G	1.86872	6.8668	9.11E-33
37	ENSG00000134419	RPS15A	1.85781	6.83867	1.37E-38
38	ENSG00000141682	PMAIP1	1.85673	6.02808	2.72E-28

39	ENSG00000235174	RPL39P3	1.84731	5.91713	6.51E-29
40	ENSG00000145592	RPL37	1.8202	10.49067	9.50E-42
41	ENSG00000233016	SNHG7	1.81465	3.02129	2.70E-08
42	ENSG00000110700	RPS13	1.81288	9.94619	2.03E-36
43	ENSG00000251495	PPIAP11	1.80555	1.87756	1.13E-04
44	ENSG00000212664	AC064799.1	1.79487	1.93587	6.41E-05
45	ENSG00000008988	RPS20	1.78385	10.22783	6.16E-36
46	ENSG00000061656	SPAG4	1.76559	2.54807	9.43E-07
47	ENSG00000133112	TPT1	1.74132	9.85153	3.99E-33
48	ENSG00000068028	RASSF1	1.73965	2.19747	8.37E-05
49	ENSG00000164761	TNFRSF11B	1.73698	2.87235	1.66E-07
50	ENSG00000181826	RELL1	1.73342	2.76074	2.64E-07
51	ENSG00000071082	RPL31	1.73017	9.94803	1.80E-38
52	ENSG00000274276	CBSL	1.69739	2.35854	3.63E-03
53	ENSG00000177954	RPS27	1.67064	10.12355	4.78E-37
54	ENSG00000255717	SNHG1	1.66369	5.2712	8.67E-18
55	ENSG00000121039	RDH10	1.65994	1.84928	1.15E-03
56	ENSG00000163682	RPL9	1.65123	9.68254	1.74E-34
57	ENSG00000237550	RPL9P9	1.64282	8.97003	3.50E-25
58	ENSG00000101132	PFDN4	1.63264	6.259	5.80E-23
59	ENSG00000269293	ZSCAN16-AS1	1.61453	1.71323	1.70E-03
60	ENSG00000227615	AP001324.1	1.61181	5.39334	2.31E-17
61	ENSG00000166441	RPL27A	1.61044	10.27831	3.52E-30
62	ENSG00000182899	RPL35A	1.57163	9.72963	1.04E-29
63	ENSG00000218426	AL590867.2	1.56681	5.41114	2.68E-13
64	ENSG00000250321	AC079140.2	1.55754	2.46534	5.38E-05
65	ENSG00000108590	MED31	1.55445	1.8765	1.65E-03
66	ENSG00000114942	EEF1B2	1.54934	9.33113	1.67E-27
67	ENSG00000217716	RPS10P3	1.5483	2.18057	4.54E-04
68	ENSG00000113739	STC2	1.53535	5.79083	2.16E-21
69	ENSG00000118181	RPS25	1.53152	9.92922	4.84E-29
70	ENSG00000244128	LINC01322	1.51806	2.15835	8.85E-04
71	ENSG00000126756	UXT	1.51489	6.21119	1.19E-20
72	ENSG00000228589	SPCS2P4	1.51038	3.13171	6.36E-05
73	ENSG00000237296	SMG1P1	1.50832	2.01957	1.54E-03
74	ENSG00000229117	RPL41	1.5052	10.20359	1.83E-33
75	ENSG00000166741	NNMT	1.50171	6.76693	1.12E-25
76	ENSG00000177600	RPLP2	1.49945	9.54988	7.02E-28
77	ENSG00000185834	RPL12P4	1.49551	2.35525	2.71E-04
78	ENSG00000125691	RPL23	1.47056	10.232	6.36E-30
79	ENSG00000240036	AC104563.1	1.46506	1.8935	3.53E-03
80	ENSG00000188243	COMMD6	1.46414	6.03248	1.11E-20

81	ENSG00000263266	RPS7P1	1.45603	6.51821	1.92E-19
82	ENSG00000145425	RPS3A	1.45495	9.47059	9.71E-30
83	ENSG00000243927	MRPS6	1.453	5.69047	3.02E-16
84	ENSG00000113811	SELENOK	1.4505	5.42095	4.05E-16
85	ENSG00000166136	NDUFB8	1.4471	2.26391	9.23E-04
86	ENSG00000178381	ZFAND2A	1.44058	2.5215	2.28E-04
87	ENSG00000137955	RABGGTB	1.43937	6.13734	6.34E-18
88	ENSG00000240395	RPL5P23	1.43419	2.27483	8.46E-04
89	ENSG00000237991	RPL35P1	1.43052	2.08439	2.15E-03
90	ENSG00000149196	HIKESHI	1.43032	6.10318	1.92E-12
91	ENSG00000119048	UBE2B	1.43026	5.60463	9.95E-15
92	ENSG00000120833	SOCS2	1.42946	5.95166	1.40E-17
93	ENSG00000172809	RPL38	1.42837	9.19553	3.48E-25
94	ENSG00000007255	TRAPPC6A	1.42473	2.23321	2.77E-03
95	ENSG00000170222	ADPRM	1.42331	2.57118	7.50E-04
96	ENSG00000244313	AC024293.1	1.42217	6.51997	1.68E-15
97	ENSG00000230453	ANKRD18B	1.41729	1.69466	1.99E-02
98	ENSG00000235508	RPS2P7	1.4171	1.98911	3.66E-03
99	ENSG00000111696	NT5DC3	1.41705	1.81656	5.60E-03
100	ENSG00000162599	NFIA	1.41609	2.09689	1.73E-03
101	ENSG00000111907	TPD52L1	1.41509	1.93619	4.14E-03
102	ENSG00000164919	COX6C	1.41497	5.87041	3.40E-17
103	ENSG00000214784	AC010468.1	1.41233	2.19936	1.63E-03
104	ENSG00000226259	GTF2H2B	1.41138	2.38165	9.82E-04
105	ENSG00000135919	SERPINE2	1.4085	5.86105	4.22E-16
106	ENSG00000095303	PTGS1	1.40545	2.38803	1.06E-03
107	ENSG00000184924	PTRHD1	1.39787	2.39369	9.07E-04
108	ENSG00000234797	RPS3AP6	1.37828	5.74887	1.40E-15
109	ENSG00000164136	IL15	1.37442	2.0254	4.23E-03
110	ENSG00000279483	AC090498.1	1.37263	2.01407	1.76E-02
111	ENSG00000168894	RNF181	1.35895	5.9266	2.68E-16
112	ENSG00000226360	RPL10AP6	1.35773	2.84243	1.90E-04
113	ENSG00000247516	MIR4458HG	1.35396	2.63236	4.39E-04
114	ENSG00000142676	RPL11	1.35096	10.04078	7.02E-25
115	ENSG00000212802	RPL15P3	1.35081	6.56595	1.28E-17
116	ENSG00000131979	GCH1	1.34895	2.59808	4.21E-04
117	ENSG00000143543	JTB	1.34357	2.91921	9.11E-05
118	ENSG00000114125	RNF7	1.34337	5.43317	6.12E-13
119	ENSG00000171223	JUNB	1.34317	2.8867	1.39E-04
120	ENSG00000183298	RPSAP19	1.33368	6.05997	1.06E-14
121	ENSG00000171863	RPS7	1.33307	9.54279	1.34E-20
122	ENSG00000254612	AP001000.1	1.32937	3.10942	1.98E-04

123	ENSG00000170889	RPS9	1.31168	9.97012	4.94E-21
124	ENSG00000163882	POLR2H	1.31154	6.30448	5.97E-15
125	ENSG00000175193	PARL	1.30918	2.46662	1.90E-03
126	ENSG00000109099	PMP22	1.30715	2.9155	1.89E-04
127	ENSG00000117877	CD3EAP	1.30521	2.1912	6.52E-03
128	ENSG00000176171	BNIP3	1.30217	6.23416	2.21E-11
129	ENSG00000167550	RHEBL1	1.29991	2.55841	2.41E-03
130	ENSG00000242951	AC007182.2	1.29986	2.51509	2.38E-03
131	ENSG00000138326	RPS24	1.29575	10.05311	1.76E-21
132	ENSG00000180211	FO393411.1	1.29532	2.64156	8.97E-04
133	ENSG00000180354	MTURN	1.29087	2.96072	3.26E-04
134	ENSG00000107833	NPM3	1.28423	6.30854	3.02E-16
135	ENSG00000249859	PVT1	1.27954	2.93605	1.01E-03
136	ENSG00000216866	RPS2P55	1.27862	2.83869	6.03E-04
137	ENSG00000178458	H3F3AP6	1.27688	2.73159	1.01E-03
138	ENSG00000114391	RPL24	1.27331	9.64454	6.57E-19
139	ENSG00000229644	NAMPTP1	1.2715	2.26877	5.56E-03
140	ENSG00000198625	MDM4	1.26611	2.54466	1.61E-03
141	ENSG00000171421	MRPL36	1.26329	5.62336	7.41E-11
142	ENSG00000153822	KCNJ16	1.26086	2.22576	7.26E-03
143	ENSG00000181061	HIGD1A	1.25942	6.47214	8.20E-14
144	ENSG00000198856	OSTC	1.25425	6.48572	3.57E-15
145	ENSG00000138166	DUSP5	1.25098	5.8367	2.68E-13
146	ENSG00000185088	RPS27L	1.25038	5.60456	8.32E-11
147	ENSG00000117691	NENF	1.24792	6.48416	5.73E-17
148	ENSG00000166710	B2M	1.24734	9.19999	1.01E-20
149	ENSG00000164615	CAMLG	1.24663	6.0483	9.80E-10
150	ENSG00000004779	NDUFAB1	1.2354	5.70947	2.49E-10
151	ENSG00000139289	PHLDA1	1.23143	6.43166	1.87E-16
152	ENSG00000185022	MAFF	1.23022	2.91175	4.94E-04
153	ENSG00000235912	AL031729.1	1.229	2.09241	1.25E-02
154	ENSG00000213189	BTF3L4P2	1.22828	2.69774	3.20E-03
155	ENSG00000100387	RBX1	1.2269	6.50946	5.55E-14
156	ENSG00000100292	HMOX1	1.22427	6.60971	3.51E-16
157	ENSG00000104231	ZFAND1	1.22356	5.48885	1.68E-10
158	ENSG00000229180	AC006001.3	1.22305	2.95285	6.05E-04
159	ENSG00000198818	SFT2D1	1.21872	5.92082	2.55E-12
160	ENSG00000015133	CCDC88C	1.21726	2.45585	4.94E-03
161	ENSG00000125726	CD70	1.21671	5.62274	3.30E-11
162	ENSG00000224631	RPS27AP16	1.21467	5.54977	5.66E-10
163	ENSG00000198258	UBL5	1.21187	6.53833	7.09E-16
164	ENSG00000166295	ANAPC16	1.21083	5.84694	1.06E-13

165	ENSG00000109220	CHIC2	1.20546	2.11081	1.61E-02
166	ENSG00000178464	RPL10P16	1.19782	5.61995	6.72E-10
167	ENSG00000128699	ORMDL1	1.19433	5.52126	2.41E-11
168	ENSG00000217130	AL139100.1	1.19389	2.6846	5.35E-03
169	ENSG00000117543	DPH5	1.19086	5.81231	1.10E-10
170	ENSG00000166595	FAM96B	1.18792	5.96022	8.32E-12
171	ENSG00000136942	RPL35	1.17717	9.6819	2.87E-15
172	ENSG00000114023	FAM162A	1.17514	5.54065	9.87E-10
173	ENSG00000149716	ORAOV1	1.16802	2.45022	8.43E-03
174	ENSG00000161970	RPL26	1.16646	5.49443	3.06E-10
175	ENSG00000244716	BX679664.3	1.16576	6.45259	4.92E-12
176	ENSG00000161980	POLR3K	1.16329	2.82673	2.36E-03
177	ENSG00000189068	VSTM1	1.16302	2.81974	7.10E-03
178	ENSG00000166002	SMCO4	1.1624	1.96861	2.48E-02
179	ENSG00000090266	NDUFB2	1.16159	5.8288	7.87E-12
180	ENSG00000137970	RPL7P9	1.16124	6.28687	2.05E-11
181	ENSG00000138085	ATRAID	1.15573	5.62013	6.68E-10
182	ENSG00000100814	CCNB1IP1	1.15523	5.64529	1.69E-09
183	ENSG00000165996	HACD1	1.15131	2.64086	5.51E-03
184	ENSG00000181524	RPL24P4	1.14999	6.01147	4.00E-13
185	ENSG00000165264	NDUFB6	1.14871	5.70898	5.34E-09
186	ENSG00000117569	PTBP2	1.14821	2.37142	1.18E-02
187	ENSG00000090263	MRPS33	1.14404	6.12557	2.34E-11
188	ENSG00000167397	VKORC1	1.14363	2.60412	5.33E-03
189	ENSG00000253683	AC027309.2	1.1409	2.56218	7.97E-03
190	ENSG00000169740	ZNF32	1.13459	2.77519	4.05E-03
191	ENSG00000136888	ATP6V1G1	1.13078	6.54966	2.80E-13
192	ENSG00000104432	IL7	1.12846	1.86251	4.60E-02
193	ENSG00000226210	AC215219.1	1.12828	2.65721	9.17E-03
194	ENSG00000233762	AC007969.1	1.12741	5.83028	3.07E-11
195	ENSG00000143256	PFDN2	1.12564	5.77719	1.69E-10
196	ENSG00000131871	SELENOS	1.12534	5.49809	6.64E-10
197	ENSG00000142188	TMEM50B	1.1253	6.2566	7.96E-13
198	ENSG00000129235	TXNDC17	1.124	6.54108	2.95E-11
199	ENSG00000175305	CCNE2	1.124	2.12231	2.53E-02
200	ENSG00000119820	YIPF4	1.12291	5.67827	4.30E-10
201	ENSG00000217241	CBX3P9	1.12166	2.12514	2.72E-02
202	ENSG00000198755	RPL10A	1.12078	9.63095	2.49E-17
203	ENSG00000276023	DUSP14	1.11736	5.47386	3.79E-09
204	ENSG00000134049	IER3IP1	1.11621	5.64982	3.84E-10
205	ENSG00000237506	RPSAP15	1.11605	5.39099	2.87E-07
206	ENSG00000164405	UQCRQ	1.116	6.47321	2.28E-12

207	ENSG00000175309	PHYKPL	1.1077	2.79837	1.55E-02
208	ENSG00000225178	RPSAP58	1.10524	6.22263	1.29E-10
209	ENSG00000183527	PSMG1	1.10443	5.75964	8.98E-09
210	ENSG00000034713	GABARAPL2	1.09819	5.88266	6.21E-10
211	ENSG00000171735	CAMTA1	1.09706	5.71952	4.07E-09
212	ENSG00000233270	SNRPEP4	1.09512	2.20507	2.77E-02
213	ENSG00000119013	NDUFB3	1.09443	6.09524	9.70E-08
214	ENSG00000105829	BET1	1.09182	5.6695	1.06E-08
215	ENSG00000175197	DDIT3	1.09054	2.08942	4.31E-02
216	ENSG00000268942	CKS1BP3	1.08965	1.96215	4.95E-02
217	ENSG00000120910	PPP3CC	1.08946	2.95951	7.03E-03
218	ENSG00000214253	FIS1	1.08864	5.8593	1.81E-09
219	ENSG00000240342	RPS2P5	1.08788	9.65924	1.11E-13
220	ENSG00000092841	MYL6	1.08669	9.11725	2.48E-12
221	ENSG00000184227	ACOT1	1.08628	1.96785	4.45E-02
222	ENSG00000186468	RPS23	1.08262	6.41027	4.76E-13
223	ENSG00000134153	EMC7	1.0787	5.69171	4.38E-08
224	ENSG00000169684	CHRNA5	1.07745	2.43018	1.80E-02
225	ENSG00000179046	TRIML2	1.07489	2.66131	2.58E-02
226	ENSG00000088451	TGDS	1.0734	3.00841	3.55E-03
227	ENSG00000249264	EEF1A1P9	1.07109	2.10823	3.11E-02
228	ENSG00000172428	COPS9	1.0704	5.4273	2.73E-07
229	ENSG00000099337	KCNK6	1.06923	2.62179	1.52E-02
230	ENSG00000132773	TOE1	1.06564	2.86968	5.72E-03
231	ENSG00000118680	MYL12B	1.06473	9.21421	8.65E-16
232	ENSG00000092208	GEMIN2	1.0645	2.79241	1.03E-02
233	ENSG00000134758	RNF138	1.06391	5.61266	9.55E-09
234	ENSG00000183696	UPP1	1.0626	6.58322	2.02E-11
235	ENSG00000214485	RPL7P1	1.05909	6.41836	1.55E-10
236	ENSG00000166342	NETO1	1.05562	2.23799	3.07E-02
237	ENSG00000115738	ID2	1.05474	2.05982	4.53E-02
238	ENSG00000153879	CEBPG	1.0533	5.62603	3.47E-09
239	ENSG00000112110	MRPL18	1.04919	6.4726	3.86E-10
240	ENSG00000122884	P4HA1	1.04842	5.74025	3.54E-08
241	ENSG00000123131	PRDX4	1.04005	6.5192	5.74E-10
242	ENSG00000160131	VMA21	1.03987	5.91528	3.36E-10
243	ENSG00000167863	ATP5PD	1.03919	6.51217	3.15E-12
244	ENSG00000143727	ACP1	1.03909	6.11405	1.11E-09
245	ENSG00000146425	DYNLT1	1.03887	5.54414	6.24E-08
246	ENSG00000146386	ABRACL	1.0332	5.94265	1.34E-08
247	ENSG00000074935	TUBE1	1.03314	2.89581	9.83E-03
248	ENSG00000126709	IFI6	1.03175	2.61149	1.42E-02

249	ENSG00000163923	RPL39L	1.03134	3.00706	6.54E-03
250	ENSG00000186184	POLR1D	1.02985	5.80849	7.70E-08
251	ENSG00000181634	TNFSF15	1.02836	2.90221	8.99E-03
252	ENSG00000148444	COMMD3	1.02745	2.5636	3.59E-02
253	ENSG00000164885	CDK5	1.02725	5.51204	9.56E-07
254	ENSG00000242071	RPL7AP6	1.02348	5.81947	4.01E-08
255	ENSG00000147687	TATDN1	1.02316	5.45534	2.21E-07
256	ENSG00000228502	EEF1A1P11	1.02164	2.4281	2.60E-02
257	ENSG00000185917	SETD4	1.02116	2.08847	4.88E-02
258	ENSG00000147669	POLR2K	1.01835	6.20378	5.55E-08
259	ENSG00000124733	MEA1	1.01535	5.48494	8.16E-07
260	ENSG00000150459	SAP18	1.01455	6.562	1.43E-10
261	ENSG00000051620	HEBP2	1.0129	6.38065	8.72E-11
262	ENSG00000000419	DPM1	1.00788	5.79092	2.72E-08
263	ENSG00000224831	TMEM183B	1.00708	2.87861	1.49E-02
264	ENSG00000172159	FRMD3	1.00567	2.71823	2.52E-02
265	ENSG00000162244	RPL29	1.00034	9.52484	7.31E-14
266	ENSG00000106701	FSD1L	1.00025	2.22818	4.72E-02
267	ENSG00000165406	MARCH8	-1.00131	5.03431	6.66E-05
268	ENSG00000077235	GTF3C1	-1.00352	5.54313	2.42E-05
269	ENSG00000175970	UNC119B	-1.00606	4.17005	3.66E-03
270	ENSG00000196935	SRGAP1	-1.00611	4.26205	4.52E-03
271	ENSG00000151208	DLG5	-1.00953	4.76984	5.42E-04
272	ENSG00000184640	SEPT9	-1.00988	7.4498	9.78E-11
273	ENSG00000115884	SDC1	-1.01142	5.32952	4.10E-05
274	ENSG00000166016	ABTB2	-1.01203	4.91491	1.67E-04
275	ENSG00000106261	ZKSCAN1	-1.0147	5.33168	2.85E-04
276	ENSG00000187239	FNBP1	-1.01695	4.08259	5.61E-03
277	ENSG00000100109	TFIP11	-1.03669	4.14906	3.53E-03
278	ENSG00000178691	SUZ12	-1.04206	5.31302	2.86E-05
279	ENSG00000130816	DNMT1	-1.04339	5.37542	1.35E-04
280	ENSG00000197386	HTT	-1.04846	5.56075	5.54E-06
281	ENSG00000138119	MYOF	-1.04949	7.35867	5.07E-08
282	ENSG00000087087	SRRT	-1.05546	5.32371	8.29E-06
283	ENSG00000064012	CASP8	-1.05857	4.34834	1.01E-03
284	ENSG00000019144	PHLDB1	-1.05942	4.30702	1.90E-03
285	ENSG00000275066	SYNRG	-1.06015	4.78132	2.59E-04
286	ENSG00000100393	EP300	-1.0637	5.41088	1.63E-04
287	ENSG00000187189	TSPYL4	-1.06397	4.26597	1.52E-03
288	ENSG00000204569	PPP1R10	-1.06485	5.18091	1.51E-05
289	ENSG00000140750	ARHGAP17	-1.06587	4.31248	1.35E-03
290	ENSG00000130589	HELZ2	-1.07397	5.35852	7.28E-06

291	ENSG00000080986	NDC80	-1.07549	4.99405	8.55E-05
292	ENSG00000143374	TARS2	-1.08204	4.63649	2.03E-04
293	ENSG00000166974	MAPRE2	-1.08601	4.23852	1.26E-03
294	ENSG00000147475	ERLIN2	-1.08873	4.86435	4.98E-05
295	ENSG00000013288	MAN2B2	-1.08904	4.53671	2.02E-03
296	ENSG00000184602	SNN	-1.09283	5.00703	1.73E-05
297	ENSG00000108557	RAI1	-1.09333	4.53819	6.08E-04
298	ENSG00000128829	EIF2AK4	-1.10052	5.09041	1.61E-05
299	ENSG00000197603	C5orf42	-1.10133	4.05225	5.60E-03
300	ENSG00000161847	RAVER1	-1.1017	4.2519	9.90E-04
301	ENSG00000085185	BCORL1	-1.10208	4.78512	2.86E-04
302	ENSG00000151929	BAG3	-1.10833	4.39114	4.67E-04
303	ENSG00000103111	MON1B	-1.11877	4.88897	3.10E-05
304	ENSG00000136861	CDK5RAP2	-1.1257	4.56652	6.55E-04
305	ENSG00000275410	HNF1B	-1.1293	5.36249	1.89E-05
306	ENSG00000198742	SMURF1	-1.13608	4.90365	2.48E-05
307	ENSG00000140320	BAHD1	-1.13873	4.13557	1.13E-03
308	ENSG00000149930	TAOK2	-1.14169	4.41958	3.94E-04
309	ENSG00000123080	CDKN2C	-1.15037	4.50798	1.71E-04
310	ENSG00000106290	TAF6	-1.15114	5.57154	1.99E-07
311	ENSG00000180340	FZD2	-1.15213	4.94552	1.17E-05
312	ENSG00000008300	CELSR3	-1.15351	4.54972	1.89E-04
313	ENSG00000088899	LZTS3	-1.15724	4.09836	1.17E-03
314	ENSG00000129116	PALLD	-1.16242	4.30348	4.91E-04
315	ENSG00000167447	SMG8	-1.17446	4.11319	8.28E-04
316	ENSG00000275832	ARHGAP23	-1.18251	5.13211	5.99E-06
317	ENSG00000204388	HSPA1B	-1.18485	4.49775	2.10E-04
318	ENSG00000100311	PDGFB	-1.19487	5.26168	1.86E-06
319	ENSG00000120800	UTP20	-1.19613	4.46581	2.16E-04
320	ENSG00000088367	EPB41L1	-1.19719	4.18602	5.94E-04
321	ENSG00000116128	BCL9	-1.1979	4.38002	2.59E-04
322	ENSG00000107882	SUFU	-1.19826	4.18662	5.63E-04
323	ENSG00000141447	OSBPL1A	-1.19979	4.86565	1.34E-05
324	ENSG00000010292	NCAPD2	-1.20468	7.94443	1.29E-14
325	ENSG00000105287	PRKD2	-1.20756	5.3405	6.51E-07
326	ENSG00000136715	SAP130	-1.21527	4.52286	6.74E-05
327	ENSG00000143624	INTS3	-1.21771	4.64168	4.28E-05
328	ENSG00000143515	ATP8B2	-1.21977	4.75258	7.34E-05
329	ENSG00000117115	PADI2	-1.22249	7.86466	1.11E-14
330	ENSG00000127585	FBXL16	-1.23025	4.85985	1.13E-05
331	ENSG00000166750	SLFN5	-1.23176	4.76255	7.02E-05
332	ENSG00000196182	STK40	-1.23676	4.21983	2.85E-04

333	ENSG00000186834	HEXIM1	-1.24389	4.70002	2.21E-05
334	ENSG00000103043	VAC14	-1.24788	4.99586	2.73E-06
335	ENSG00000076382	SPAG5	-1.25553	4.84811	7.76E-06
336	ENSG00000140829	DHX38	-1.26122	4.64039	2.00E-05
337	ENSG00000198420	TCAF1	-1.26525	4.88863	3.49E-05
338	ENSG00000070366	SMG6	-1.26997	4.44081	6.78E-05
339	ENSG00000126464	PRR12	-1.28188	5.50453	2.44E-08
340	ENSG00000061273	HDAC7	-1.28418	4.93608	4.37E-06
341	ENSG00000151458	ANKRD50	-1.29771	4.6325	1.65E-05
342	ENSG00000183853	KIRREL1	-1.30882	7.54128	2.38E-14
343	ENSG00000101298	SNPH	-1.31718	4.52499	2.01E-05
344	ENSG00000108797	CNTNAP1	-1.33676	4.25263	8.55E-05
345	ENSG00000185340	GAS2L1	-1.34087	4.54482	1.03E-05
346	ENSG00000078804	TP53INP2	-1.35485	4.41988	2.05E-05
347	ENSG00000139613	SMARCC2	-1.35593	5.37013	2.47E-07
348	ENSG00000160877	NACC1	-1.38394	4.70478	2.07E-06
349	ENSG00000113368	LMNB1	-1.39199	5.59264	5.52E-10
350	ENSG00000184675	AMER1	-1.43458	2.15215	4.20E-02
351	ENSG00000166897	ELFN2	-1.45441	5.19417	4.38E-08
352	ENSG00000101868	POLA1	-1.46027	4.30357	1.61E-05
353	ENSG00000160233	LRRC3	-1.46726	2.42858	4.82E-02
354	ENSG00000135476	ESPL1	-1.46898	5.36398	9.08E-10
355	ENSG00000085872	CHERP	-1.47329	4.5681	4.37E-06
356	ENSG00000090975	PITPNM2	-1.48735	2.44689	4.20E-02
357	ENSG00000147536	GINS4	-1.49165	2.45098	3.90E-02
358	ENSG00000085117	CD82	-1.51339	2.47129	3.45E-02
359	ENSG00000100350	FOXRED2	-1.52448	4.61088	5.51E-07
360	ENSG00000110344	UBE4A	-1.53031	4.47081	3.16E-06
361	ENSG00000148200	NR6A1	-1.54771	2.25349	2.38E-02
362	ENSG00000215256	DHRS4-AS1	-1.54796	2.50262	3.62E-02
363	ENSG00000118898	PPL	-1.55375	4.76674	2.27E-07
364	ENSG00000180884	ZNF792	-1.55657	2.2626	4.78E-02
365	ENSG00000149639	SOGA1	-1.56396	4.73389	5.84E-07
366	ENSG00000156970	BUB1B	-1.58468	4.98366	4.22E-09
367	ENSG00000273820	USP27X	-1.5898	1.98751	2.41E-02
368	ENSG00000137802	MAPKBP1	-1.64511	2.59167	1.72E-02
369	ENSG00000113209	PCDHB5	-1.65836	2.35616	3.08E-02
370	ENSG00000179104	TMTC2	-1.70977	2.40391	2.93E-02
371	ENSG00000145246	ATP10D	-1.71035	2.40435	2.13E-02
372	ENSG00000179546	HTR1D	-1.71477	2.40695	1.83E-02
373	ENSG00000124444	ZNF576	-1.73563	2.12239	4.80E-02
374	ENSG00000078177	N4BP2	-1.75069	2.14342	4.91E-02

375	ENSG00000214357	NEURL1B	-1.78157	2.165	3.65E-02
376	ENSG00000174943	KCTD13	-1.81415	2.19564	3.28E-02
377	ENSG00000185567	AHNAK2	-1.84007	7.69372	1.42E-22
378	ENSG00000188322	SBK1	-1.87249	2.55257	9.90E-03
379	ENSG00000083097	DOPEY1	-1.91288	2.29001	2.02E-02
380	ENSG00000129951	PLPPR3	-1.94746	2.31993	1.96E-02
381	ENSG00000133247	KMT5C	-1.96626	1.94471	3.69E-02
382	ENSG00000198324	FAM109A	-2.02156	2.3859	1.05E-02
383	ENSG00000169951	ZNF764	-2.04044	2.01236	2.79E-02
384	ENSG00000272405	AL365181.3	-2.0468	2.41114	7.94E-03
385	ENSG00000213763	ACTBP2	-2.07149	2.43889	3.40E-03
386	ENSG00000256087	ZNF432	-2.0834	2.05544	2.40E-02
387	ENSG00000269416	LINC01224	-2.15759	2.51166	3.81E-03
388	ENSG00000272031	ANKRD34A	-2.22547	2.18456	1.43E-02
389	ENSG00000270504	AL391422.3	-2.24214	2.2072	4.21E-03
390	ENSG00000178401	DNAJC22	-2.2493	2.2088	9.35E-03
391	ENSG00000239665	AL157392.3	-2.36185	2.3109	9.09E-04
392	ENSG00000197705	KLHL14	-2.3629	2.32217	7.83E-03
393	ENSG00000110400	NECTIN1	-2.38314	2.33714	6.01E-03
394	ENSG00000163093	BBS5	-2.38718	1.78496	7.09E-03
395	ENSG00000184545	DUSP8	-2.40733	1.80458	2.52E-02
396	ENSG00000165821	SALL2	-2.43562	2.3804	3.58E-03
397	ENSG00000171992	SYNPO	-2.44496	2.39151	3.44E-03
398	ENSG00000100592	DAAM1	-2.47066	1.86449	2.21E-02
399	ENSG00000141448	GATA6	-2.61849	2.00192	2.19E-03
400	ENSG00000007545	CRAMP1	-2.73237	2.10933	6.41E-03
401	ENSG00000135439	AGAP2	-2.80063	2.17133	8.03E-04
402	ENSG00000135338	LCA5	-3.55409	1.94867	8.20E-04
403	ENSG00000181896	ZNF101	-3.65445	2.04052	3.08E-04
404	ENSG00000133069	TMCC2	-8.98839	1.75942	1.61E-05
